# Practice and perspectives in the validation of resource management models

**DOI:** 10.1038/s41467-018-07811-9

**Published:** 2018-12-18

**Authors:** Sibel Eker, Elena Rovenskaya, Michael Obersteiner, Simon Langan

**Affiliations:** 1 International Institute for Applied Systems Analysis (IIASA), Schlossplatz 1, A2361 Laxenburg, Austria; 2 Faculty of Computational Mathematics and Cybernetics, Lomonosov Moscow State University, Moscow, Russia

## Abstract

Quantitative modelling is commonly used to assist the policy dimension of sustainability problems. Validation is an important step to make models credible and useful. To investigate existing validation viewpoints and approaches, we analyse a broad academic literature and conduct a survey among practitioners. We find that empirical data plays an important role in the validation practice in all main areas of sustainability science. Qualitative and participatory approaches that can enhance usefulness and public reliability are much less visible. Data-oriented validation is prevalent even when models are used for scenario exploration. Usefulness regarding a given task is more important for model developers than for users. As the experience of modellers and users increases, they tend to better acknowledge the decision makers’ demand for clear communication of assumptions and uncertainties. These findings provide a reflection on current validation practices and are expected to facilitate communication at the modelling and decision-making interface.

## Abstract (web summary)

Credibility of long-term projection in quantitative models is continuously under debate and they rely on validation to prove projection accuracy. Here the authors investigated the views on the validation approaches and they show that empirical data plays an important role in the validation practice in all main areas of sustainability science.

## Introduction

Quantitative modelling is an essential component of socio-environmental and economic research, management and policymaking. A broad range of models, including integrated assessment models, impact assessment models, environmental models, systems models, and so forth, address resource management problems in various areas related to sustainability, from ecosystems to energy systems. Some of these models have a prescriptive stance, generating recommendations for action. Others aid decision-making in different ways, for example with long-term projections, while capturing the complexity of various physical, economic and social factors. Standing at the intersection of environmental science, economics and decision sciences, such modelling studies have resulted in influential applications, such as the large modelling framework used by the European Commission for the impact assessment of energy and environmental policies^1–3^, or the National Energy Modelling System (NEMS) of the US Energy Information Administration^4^.

The credibility of long-term projections generated by quantitative models has been the topic of a heated debate. Several critiques in the academic literature and popular media outlets highlighted that models are typically used as if they are precise predictors, and uncertainties are ignored^5–7^; that the methods used to deal with uncertainties are often inadequate or unsuitable^8–10^; that models routinely extrapolate past data as if it is a good estimate of the future^11,12^; and that models usually have a limited scope, and often omit relevant and important processes^11,13^.

Validation is the modelling step commonly employed to deal with such criticism and to establish a sound grounding for models at the science-policy interface. The definition of validation is equivocal across different scientific fields. In decision sciences, validation usually implies establishing confidence in the model by judging its usefulness with respect to some purpose^14,15^. In environmental modelling, validity is often used to indicate that model predictions are consistent with observational data, or that the model is an accurate representation of physical reality, or both^16–18^. In this study, based on the dictionary meaning of valid as well-grounded and justifiable^19^, we use the term validation as a general process of evaluating a model’s performance and suitability for its intended use. We do not refer only to the representation accuracy, but we imply establishing confidence in the model by employing a variety of assessment tools.

These different connotations to the term validation stem from different philosophies of science. Positivism and relativism are often stated as the two distinct philosophies of science underlying the validation viewpoints^18,20,21^. Being rooted in logical empiricism, which argues that knowledge is acquired by observational data and the interpretation thereof through logic and reason, positivist validation approaches focus on an accurate representation of reality and employ statistical tests to compare the model output and the data. The positivist viewpoint on validation reflects the practice in natural sciences. Alternatively, the relativist view on validation originates from the challenge Thomas Kuhn posed on the objectivity assumption in positivism, that is, scientific knowledge is relative since it depends on the paradigm prevalent at the time of a study. The relativist validation approaches value the model usefulness, for instance in the sense of fitness for purpose, more than the representation accuracy and employ semiformal and conversational tools.

In decision sciences, economics, and management science, validation approaches cover both of these philosophical viewpoints^20,21^. Practitioners often acknowledge the value of both, and employ a combination of multiple validation techniques accordingly. In the environmental modelling domain, validation is seen more from a positivist viewpoint. An extensive body of work is devoted to developing, advancing and compiling data-oriented techniques to ensure data and behaviour validity^22–25^. Still, many studies suggest integrated frameworks that assess conceptual and methodological validity along with data and behaviour validity^26–29^, and some studies offer qualitative frameworks and participatory approaches to involve stakeholder views, and hence to enhance the extent of public trust in modelling studies^5,29–32^.

Conventional validation approaches that aim to ensure that models reflect reality with appropriate accuracy support a modelling paradigm that tries to predict a best-estimate future by reducing the uncertainty in future projections. However, they may not align well with alternative paradigms that urge a stronger acknowledgement of uncertainties in modelling, analysis and communication^7,33–37^. The modelling paradigm referred to as exploring multiple plausible futures avoids predicting a best-estimate or probabilistic future for situations where a single reliable model cannot be consolidated and appropriate probability distributions cannot be elicited^38^. In this paradigm—also named exploratory modelling^39–41^—models are used as heuristics to guide decision making. In particular, they are used in computational experiments that link various, sampling-based alternative realisations of uncertain model inputs, that is, parameters, functions or structures, to model outputs. Each computational experiment corresponds to a what-if scenario, hence the model actually generates a large ensemble of exploratory scenarios. Such scenario ensembles can be used to elaborate decision heuristics by, for example, specifying the decision objectives and searching for the most salient scenarios. Therefore, such scenario ensembles can be highly promising for future decision support^42^. In such alternative paradigms, representation accuracy plays a less important role in validation, because the representation of the system is expected to be uncertain, and validation does not aim to reduce this uncertainty.

This study investigates existing viewpoints on and approaches to general model validation practice, and when models are used specifically for scenario generation to explore multiple plausible futures. The underlying motivation is two-fold. Firstly, we expect to provide model developers and users with reflections on and insights into their practice. Secondly, the information about existing validation viewpoints and the factors that affect them can facilitate communication at the interface between modelling and decision making. With better communication, the parties can further elaborate on the requirements for a model’s validity and its contribution to decision-making.

## Results

### Viewpoints in the literature

To get an overview of the concepts and viewpoints governing the model validation practice, we examine a broad academic literature using text-mining tools. We employ two datasets of publications focusing on validation. The first one contains publications from various fields with model validation in their keywords, while the second dataset, being a subset of the first, includes scenario as a keyword, too. (See Methods section for the specification and sizes of these datasets.) The first observation on this body of literature is that the number of model validation publications has significantly increased over time, hence the weight of recent publications in our dataset is higher. However, only a small fraction (<1%) of modelling studies focus on validation explicitly regardless of time. (Supplementary Figure 1).

We identify the main topics in the abstracts of these publications by using a well-established text-mining technique called topic modelling. Performed on the two datasets specified above, topic modelling pinpoints the main concepts of validation practice, and reveals whether it is different when models are used specifically for scenario analyses.

**Fig. 1:**
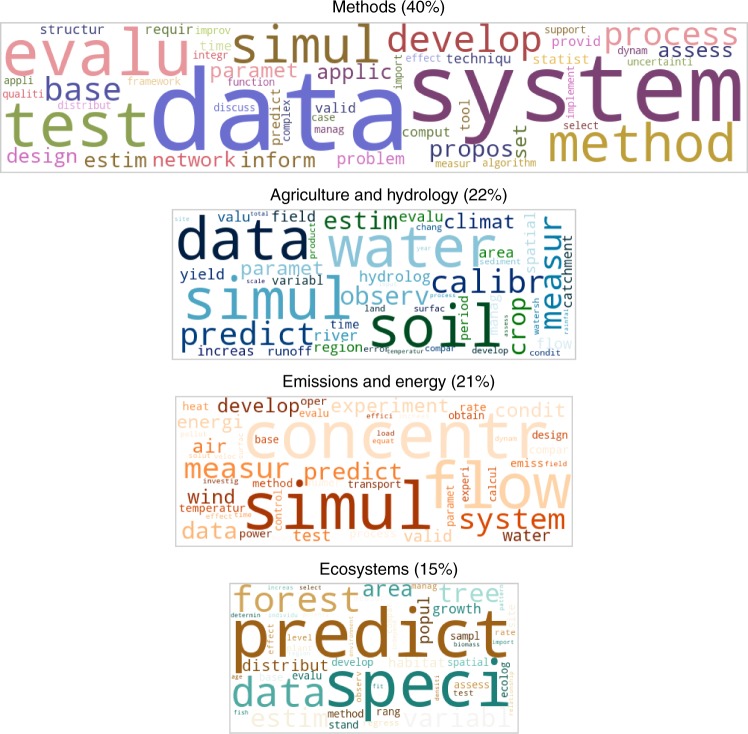
The four main topics in the validation publications The figure shows the four topics identified by the application of a topic modelling algorithm (Latent Dirichlet Allocation) on the publications in Dataset I. The word clouds depict the top 50 most frequent, hence the most descriptive words associated with each topic. The size of each cloud is respective to the fraction of publications associated with the corresponding topic. For instance, 40% of the documents fall into the topic labelled as Methods, therefore the corresponding word cloud is the largest. In each word cloud, the bigger the font of a word, the more descriptive it is for the corresponding topic. The topics are not mutually exclusive in terms of their word content. Considering their word content, one topic is concluded to be about Methods, while the other three relate to the three main areas of sustainability science. Data is a frequent word, hence a prevalent concept, in the validation publications of all four topics. Source data of this figure are provided in Supplementary Data 1

Four main topics emerge from the first dataset: three application areas and a topic covering general methodological subjects (Fig. 1). Attributed to frequent words such as flow, concentration, energy, power and air, the first application area covers a combination of Emissions and Energy studies. Data and measurement are frequently mentioned concepts in these studies, indicating a strong emphasis on empirical data in validation. The second application area covers a combination of Agriculture and Hydrology studies, as indicated by the words water, runoff, sediment, soil and crop. Data and prediction are frequent concepts, and calibration stands out in this topic differently from the other topics. The third topic refers to Ecosystems studies as indicated by common words such as forest, species, and habitats. The words predict and data are very frequent in this topic, which can be interpreted as a prediction-orientation in modelling, and the importance of data in validation. The general methodological subjects are grouped into another topic. Apart from a strong emphasis on a systems view in the Methods topic, data emerges as a highly prominent concept, alongside words such as parameter, estimate, and statistics. Together, these indicate a validation viewpoint focused on formal and data-oriented techniques.

**Fig. 2:**
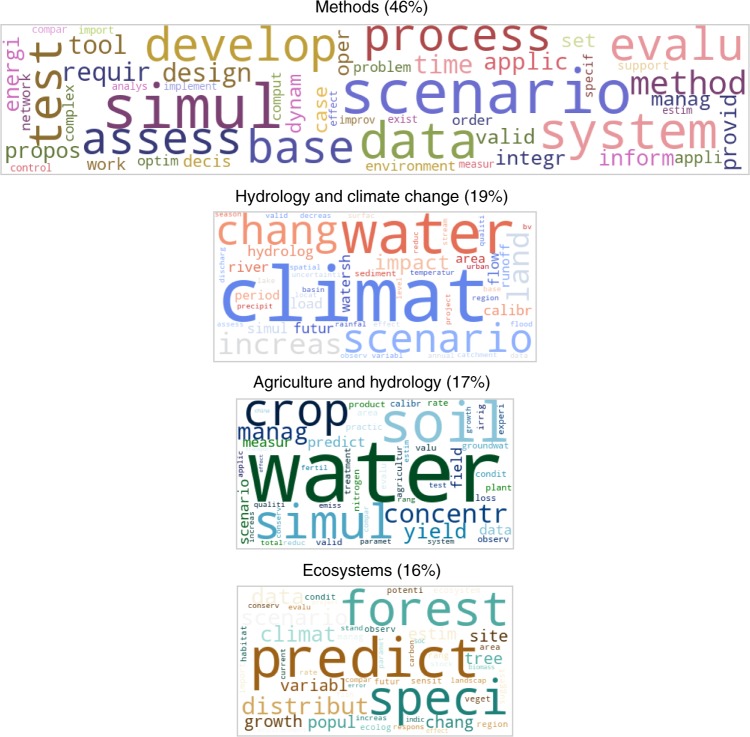
The four topics in the validation and scenario publications. The figure shows the four topics identified by the application of a topic modelling algorithm (Latent Dirichlet Allocation) on the publications in Dataset II, which contains the scenario-oriented studies in particular in addition to model validation. The word clouds depict the top 50 most frequent, hence the most descriptive words associated with each topic. The size of each cloud is respective to the fraction of publications associated with the corresponding topic. In each word cloud, the bigger the font of a word, the more descriptive it is for the corresponding topic. Considering the word content, the Methods topic focuses on simulation, (model) development, evaluation and testing. Data is a descriptive word again, and uncertainty is not among the top fifty words, although it is expected to be an important concept in scenario studies. The other three topics again refer to the main areas of sustainability science. Scenario approaches are more apparently associated with Hydrology and Climate Change studies, while the Ecosystems studies emphasise prediction. Source data of this figure are provided in Supplementary Data 1

Considering model validation in the context of scenario studies, one of the four topics identified relates to the methodological aspects of these studies as before, while the others cover the application areas (Fig. 2). The largest portion of the publications is associated with the Methods topics, which includes terms such as simulation, evaluation, and testing. Data is a descriptive word in this topic, yet not as strongly emphasised as in the general modelling concept. Uncertainty is not among the top fifty words in this Methods topic, although it is expected to be an important concept in scenario studies. This finding can be related to the dominant uncertainty framing attitudes in scientific publications, which are shown to be expressing the findings as facts and defending the results based on a validation^43^, without an explicit discussion of uncertainty. Among the scenario-focused publications, Agriculture and Hydrology studies are distinguished from Hydrology and Climate Change studies, where the latter is defined by frequent words such as climate change, land, scenario and future. This finding indicates that the scenario approach to investigate the effects of climate change is more common in hydrology studies than in agriculture and ecosystems studies. The Ecosystems topic, defined by frequent words such as forest and species, has a strong emphasis on prediction, which do not appear among the top words in other topics.

This analysis of a large body of academic literature shows a prediction-orientated modelling and a strong emphasis on empirical data, aligning with the representativeness viewpoint. Such approaches are very common even in the studies that involve scenario analyses, while uncertainty is scarcely mentioned. Still, in the scenario-oriented studies, the emphasis on data and prediction is not as strong as it is in the general modelling studies.

### Viewpoints of practitioners

We complement the general information derived from text mining of academic papers with the current validation viewpoints and approaches among modelling practitioners. We employ a short online survey circulated among researchers and policy analysts in academia, policy organisations and industry. Following a clarification about the modelling context, the survey contained a series of Likert scale questions about validation in general, and in the context of scenario generation in particular. The respondents were also asked to report their background, such as the type of organisations they have worked at, their experience with modelling and their modelling role (Supplementary Figure 2), the modelling areas they have worked on (Supplementary Figure 3), and the validation techniques they have used (Supplementary Figure 4). Below we discuss the responses to the Likert scale questions, and their relation to the background factors, if there is a statistically significant dependence.

Validation in the general modelling context: The survey questions on model validation primarily address the representativeness and usefulness views on validation. The representativeness view is based on positivism, yet a purely positivist validation based on observational data is argued to be impossible^17,18,44^. Reasons given to support this argument first include that multiple models can generate the same output as the equifinality principle implies; therefore, there is no uniquely true model that can fit to empirical data^20,44^. Secondly, there is no guarantee that a model can successfully project the future if it can replicate the past, because modelling assumptions like scaling up, averaging, and reducing the resolution level can cause deviations in future projections, even though they replicate the empirical data in a given spatial and temporal scale^45^. Furthermore, an objective validation cannot be expected when both the assessment of a fit between the model and empirical data, and the measurement of the data itself is inference-laden^17^. Alternative formal validation approaches have been developed to deal with such problems, for instance the generalised likelihood uncertainty estimation (GLUE) method^46,47^ that addresses equifinality and accepts multiple models as valid based on statistical inference. However, the use of such approaches have remained limited to producing uncertainty intervals around the average model output^48^.

The impossibility of an accurate representation and projection leads to the usefulness view in validation, where usefulness can be defined as how well a model fits for a given purpose. Many scholars object to using models for prediction in the first place, and argue that they should rather be used as heuristics to enhance understanding and guide decision-making^44,49,50^. Policy problems require a comprehensive critique of the scientific enquiry rather than a purely rationalist one, for instance to include practical and ethical concerns^51^. Therefore, using models as heuristics can provide a broader and multidimensional view on policy problems. It can assist the formulation of alternative policies which can deal with various and often unexpected situations. It also allows experimenting with different value systems of stakeholders, especially in participatory settings with citizens where models are used as metaphors to identify implicit norms affecting a policy problem^52^. Therefore, this way of using models has potential implications for consensus building. A model can be used in several other alternative ways, from data condensing to training users for a particular behaviour^49^. Therefore, a model that can provide benefits for any of such purposes would be considered useful.

These fundamental issues have been discussed for decades, yet they are still the main topics of debate, especially for models that cannot be limited to physical systems and a natural sciences perspective due to the involvement of human and decision-making factors. Therefore, the dichotomy between representativeness and usefulness, the role of empirical data in validation, and the view of decision-makers on validity, are the key dimensions we consider while investigating the existing viewpoints on validation.

**Fig. 3:**
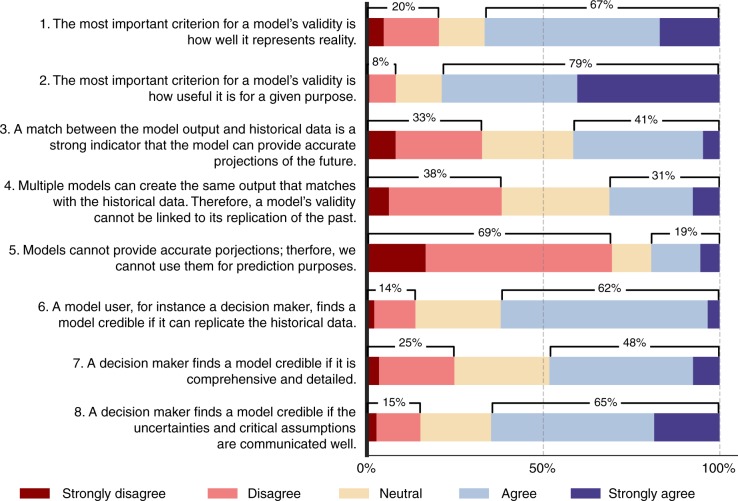
Survey responses to the key issues in model validation. The figure shows the responses given to the survey questions about the key issues in model validation, such as the validity criterion (Question 1 and 2), the role of historical data (Questions 3–5) and the decision-makers’ view (Questions 6–8). The length of the bars refer to the fraction of responses given to each question on the Likert scale from Strongly disagree to Strongly agree. The majority of respondents consider both the representation of reality and usefulness as important validity criteria, support the usability of models for prediction purposes, and acknowledge the decision-makers’ demand for transparency. Source data of this figure are provided in Supplementary Data 1

Survey responses (*n* = 164) show that practitioners value both the usefulness and representativeness of a model. Seventy-nine percent of the respondents agree or strongly agree that the most important validity criterion is usefulness, while 67% think that it is representativeness (Questions 1 and 2 in Fig. 3). Most respondents agree or strongly agree with both statements simultaneously (Supplementary Figure 5). Therefore, a dichotomy does not exist among practitioners. This tendency to agree with both statements does not differ among the experience levels or organisational backgrounds. As for the modelling roles, model developers and respondents who identified themselves as both developers and users tend to agree that usefulness is the most important validity criterion. Yet, model users mostly remain neutral or disagree with this statement (Supplementary Figures 6, 7 and 8). This finding is counterintuitive, considering that model users, either in research or decision-making contexts, would be expected to value how well the model serves for its purpose and favour usefulness more than representation accuracy, unless their purpose is an accurate representation. This asymmetry between the expectations of modellers and model users has been noted earlier, and ascribed to the lack of information non-modellers have about the limitations in models, hence their higher demands for representation accuracy^26^.

Regarding the role of historical data in validation, there is no consensus among the respondents. Forty-one percent of the respondents consider the reproducibility of the past data a strong evidence of model validity for providing accurate future projections, while 33% disagree with this statement (Question 3). About one third of the respondents agree and another one third disagree that validity cannot be linked to the replication of the past since multiple models can achieve this (Question 4), indicating an absence of consensus about the equifinality principle in validation. A large majority (69%) responds negatively to Question 5, implying that they support the usability of models for prediction purposes. These viewpoints about the role of data are not dependent on the background of the respondents. Overall, these findings indicate that objections to relying on data-oriented validation approaches due to the impossibility of a purely positivist validation have not been widely reflected on the practice.

Concerning the view of decision-makers, e.g., the clients of models, on model validity (Questions 6–8), most respondents (62%) think that decision-makers find a model credible if it replicates the historical data, and if the assumptions and uncertainties are communicated clearly (65%). Decision-makers’ interest in comprehensiveness and detailedness of the model receives a weaker, yet significant acknowledgement (48%). Therefore, practitioners think that data-driven validation is demanded by decision-makers, and they acknowledge the call for clarifying uncertainties and assumptions, which can be considered as best-practice in contemporary modelling. The acknowledgement of the communication of uncertainties and assumptions depends on experience level. Surprisingly, more of the respondents with moderate experience (2–10 years) disagree with this statement, compared to the respondents with very short and long experience (Supplementary Figure 9). This finding can be interpreted as follows: A longer engagement in modelling and a longer interaction with decision-makers help to acknowledge the necessity of communicating uncertainties and assumptions regardless of the frustrations it may cause. As for the high support of less experienced respondents, it can be attributed to fresh training on the best-practice of modelling. The employment conditions, which are beyond the scope of this paper, may play a role, too.

Validation in the scenario generation context: When the models are used for scenario generation, the focus shifts from the model to the broader analytical context. Therefore, validation in exploratory modelling is suggested to consider the reasonability of modelling assumptions, the strategy of sampling to generate the scenarios, and the logic of connecting experimental results to policy recommendations^53^. Yet, when new models are developed for exploring multiple plausible futures, the reported validation techniques are similar to those used in a general modelling context. Comparison of model output for a single baseline scenario to historical data remains the most commonly used technique, while extreme conditions tests, cross-validation and reality cheques are also employed^54–56^. Sensitivity analysis^57^ is a commonly used validation technique in general, even in participatory settings^7^. It investigates how robust the model output is against the uncertainty in inputs and identifies the factors to which the model is most sensitive. In the modelling cycle, these factors are suggested to be recalibrated for a higher accuracy, if such a sensitivity is not expected in real life^15,58^. However, this way of using sensitivity analysis in validation can reduce the model’s ability to generate a variety of scenarios if it is used for exploratory models, since it aims to make the models robust.

**Fig. 4:**
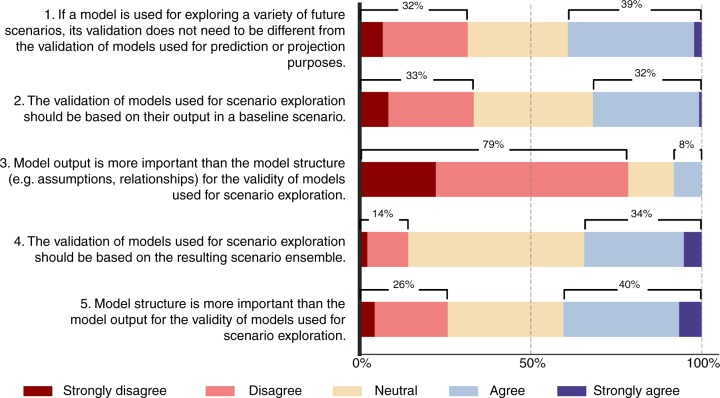
Survey responses to validation in the scenario context. The figure shows the responses given to the survey questions about the key issues in model validation in the scenario generation context. These questions cover whether validation should be different in this context than the general modelling context (Question 1), whether the validation should be based on a baseline scenario or the scenario ensemble (Questions 2 and 4), and whether the model output or structure is more important in validation (Questions 3 and 5). The length of the bars refer to the fraction of responses given to each question on the Likert scale from strongly disagree to strongly agree. There is no consensus among the respondents about these questions. Still, a large majority of the respondents disagree or strongly disagree that model output is more important than the structure in the validation of scenario-oriented model. Source data of this figure are provided in Supplementary Data 1

Using models to explore multiple plausible futures raises the question of whether models should be validated differently than the models used for prediction or projection. This is the first survey question asked to respondents in the scenario generation context. The fraction of respondents who think that validation does not need to be different is 39%, while the ones who favour a different validation approach are 32%. (Question 1 in Fig. 4). There is no strong statistical evidence for the dependence of the responses to this question on respondent characteristics. Still, experience level plays a potentially important role. The respondents with medium experience may tend to disagree with this statement more. That is, they favour a different validation approach for the scenario context (Supplementary Figure 10).

The other two questions address whether the models used for scenario generation should be validated based on a baseline scenario (Question 2) as in data-driven validation, or based on the scenario ensemble (Question 4), following a brief information about the terms ‘baseline scenario’ and ‘scenario ensemble’. 32 and 33% of respondents agree and disagree, respectively, with validation based on a baseline scenario, indicating discordant views. The scenario ensemble option receives 34% agreement, much higher than disagreement (14%). Still, most respondents are neutral about the use of scenario ensembles in validation, which may be due to unfamiliarity or ambiguity of the concept. The last two questions compare the importance of model output and structure for validation (Questions 3 and 5). Seventy-nine percent of respondents disagree with the relative importance of model output, therefore it can be said that the respondents do not favour an output-focused validation over a structure-focused one when the model purpose is scenario generation. Most respondents who disagree with the output being more important agree or remain neutral about the structure being more important (Supplementary Figure 11). Therefore, most respondents prefer to focus on the structure rather than the output in the validation of scenario-oriented models, while a smaller yet considerable group of respondents do not report such a strong preference.

## Discussion

This study investigated the viewpoints on validation in the general modelling context and in the particular scenario exploration context. Three key dimensions were considered in the general modelling context: the historical dichotomy between representativeness and usefulness, the role of empirical data in validation, and the view of decision-makers on validity. In the scenario exploration context, whether validation should be performed differently, the relative importance of model structure and output, and whether validation should be based on a baseline scenario or a scenario ensemble were the three aspects investigated.

Regarding the role of empirical data in validation, a model’s ability to replicate the past is a commonly used validity criterion, which is assumed to indicate how well the model represents reality. Many scholars have argued that data-oriented validation is not sufficient. Conceptual aspects and participatory approaches should be integrated into validation to enhance the public reliability of models. Our text-mining results do not indicate a wide implementation of this view, since the prevalent concepts in academic publications centre around data. Data plays a prominent role in validation practice in all main areas of sustainability science, including hydrology, ecosystems, emissions and energy. This emphasis on data is not specific to academic publications. Practitioners report that data comparison is one of the most commonly used techniques, and a match between the model output and data is a reliable indicator of its predictive power. Quantifying models with reliable data and checking the plausibility of the output with respect to the past data is surely an indispensable component of validation. Such a data match being seen as a reliable indicator of predictive power can be interpreted as a low acceptance of an integrated validation viewpoint.

Data-oriented validation is linked with the representativeness view on validity. Still, practitioners value the usefulness of a model as much as its representation of reality. The usefulness view does enjoy as much support as one might expect in validation practice. The reason could be the relative difficulty of defining and measuring usefulness compared to representativeness, the prevailing perception of models as descriptions of the reality rather than representations, or the absence of resources to engage experts, stakeholders and decision makers in the validation practice. Still, we echo the calls for integrated validation approaches^27^ that evaluate the conceptualisation, structure and behaviour of a model with respect to its predefined purpose, based not only on in-house testing but also on peer reviews by experts and stakeholders. We also stress the importance of publishing such validation practices explicitly to enhance visibility.

The survey aimed to investigate what practitioners think about the decision-makers’ view on validity, because models should meet the decision-makers’ expectations in order to assist decision-making. According to the perceptions of survey respondents, which are mostly scientists, decision-makers expect a model to replicate historical data, to be comprehensive and detailed, and the assumptions and uncertainties to be communicated clearly. Therefore, the emphasis on replicating the past and representing reality accurately is attributed also to the demands of decision-makers. This finding is intriguing, since it is the scientists who are often claimed to pursue scrutiny in data-intense, comprehensive and detailed models and to ignore the social and institutional complexities of decision problems. Also, the call for using small and exploratory models to support policy analysis instead of large and consolidative ones^39^ is not decision-makers’ demand, according to most survey respondents. Therefore, a closer look at the science-policy interface in future research can illuminate whether these findings are due to a perception gap between the modellers and decision-makers.

Our survey findings showed that the views on many aspects of model validity and validation are diverse, despite some convergence, for instance, on usefulness as a validity criterion. The team members of a modelling project, whether from the science or policy side, cannot be expected to have a default mutual understanding about validity and mutual expectations from the model. Therefore, we recall the importance of establishing a common understanding about what is expected from the model, and how it is to be validated.

In the scenario generation context, using models to explore multiple plausible futures instead of predicting a best-estimate future can still be considered a niche, since the academic literature vastly emphasises prediction, and most practitioners think that models can be used for prediction purposes. This view might be reflected on the survey results, since more respondents remained neutral, possibly due to unfamiliarity or ambiguity, about the questions in the scenario context than about the ones in the general context. Furthermore, the data-oriented validation approaches used for prediction-focused models are also commonly used in the scenario generation context. Still, model output is not considered more important than model structure in this context, as indicated by survey responses. The relative importance of model structure received more agreement. Therefore, model structure can be a point of departure for future validation studies in the scenario generation context.

Future research can also focus on the development of validation frameworks for scenario-oriented models. Tests like historical data comparisons or reality cheques ensure that the model successfully generates one plausible future, or that the model is structurally reasonable. Therefore, they should surely remain in such a validation framework. However, as discussed earlier, the strategy of sampling to generate the scenarios, and the logic of analytical framework that links experimental results to policy recommendations are important in validating exploratory models, too.

Whether they are generated by a model or not, scenarios are evaluated on attributes like plausibility^59^, consistency^60,61^ and diversity^62^. Therefore, model validation should include an appreciation of these attributes in the case where the scenario ensemble is generated by an exploratory model. In other words, to evaluate an exploratory model based on its purpose, i.e., generating scenarios, the plausibility, consistency and diversity attributes of its output should become part of the model assessment criteria. In practice, the relation of these attributes to the sampling strategy and the analytical framework can be elaborately defined, and then formal techniques can be developed to evaluate a model with respect to these criteria.

## Methods

### Text mining analysis

We examine a broad academic literature with text-mining tools to understand the concepts and relationships governing the model validation practice. This text-mining approach is based on the author-specified keywords, and the frequency of words in the abstracts of the publications, as it will be explained in detail below. In other words, we aim to understand the validation practice based on the words used in the publications. This text-mining technique allows covering a large number and a wide variety of publications in our analysis. It provides an impression of the prevalent concepts and major clusters of work. Yet, being based on simultaneous occurrence of words, it is not expected to reveal the exact validation approaches and methods used in the literature.

Our analysis focuses on two datasets retrieved from the Scopus database, with the motivation to investigate if there are any differences in the validation approaches when models are used specifically for scenario analysis. Dataset I contains 10,739 publications mainly from the environmental and decision sciences that address the subject of model validation. Dataset II is a subset of the first one, containing 748 publications and including scenario as a keyword besides model validation. Table 1 shows the specifications of these datasets, that is, search criteria on the Scopus database.

**Table 1:** Specifications of the datasets analyzed by text mining

	Dataset I	Dataset II
Any of the title, abstract or keywords include	“Model validation” OR “model validity” OR “model evaluation” OR “model assessment” OR “model testing”	(“model validation” OR “model validity” OR “model evaluation” OR “model assessment” OR “model testing”) AND scenario
Years	1980-present	1980-present
Language	Only English	Only English
Predefined Scopus fields^a^	▪ Environmental science ▪ Computer science ▪ Agricultural and biological sciences ▪ Mathematics ▪ Energy ▪ Social sciences ▪ Economics, econometrics and finance ▪ Decision sciences ▪ Multidisciplinary	▪ Environmental science ▪ Computer science ▪ Agricultural and biological sciences ▪ Mathematics ▪ Energy ▪ Social sciences ▪ Economics, econometrics and finance ▪ Decision sciences ▪ Multidisciplinary
Number of documents returned	10,739	748
Number of documents analysed	10,688	748

^a^Obtained by excluding all other fields, meaning that if an article is classified both in, for example, environmental science and chemistry, it is not included in this study

To identify the main concepts and themes in the literature, we employed a text-mining tool called topic modelling^63^. We adopt the most commonly used topic modelling method, which is Latent Dirichlet Allocation (LDA)^64^. In an LDA implementation, the user specifies the number of topics (bags), and then the algorithm probabilistically allocates each document to one of these bags to a certain extent. This extent signifies the topic probability of a document, and depends on the frequency of a document’s words in each of these topics. In other words, the topics are not necessarily mutually exclusive in terms of the documents and words they include. Resulting from this process, LDA forms document-topic and topic-word pairs based on the words included in each document.

The results in this paper discuss the topic contents based on the topic-word pairs. The topics are named based on the most defining, i.e., the most frequent, words in them. For instance, if a topic has water, soil, crop among the 50 most frequent words visualised in a word cloud, we name it Agriculture and Hydrology topic. The topics include many words in common, yet such common words occur in them with different probabilities and with a different set of neighbouring words. Supplementary Table 1 lists the topic probabilities of the most common words in the four topics identified for Dataset I, whereas Supplementary Table 2 includes those identified for Dataset II. These tables underlie Figs. 1 and 2, respectively. As for the document-topic pairs (Supplementary Figure 12), they show that the topics identified by LDA are quite distinct, meaning that most publications are exclusively associated with one of the topics.

For the preparation of data, we removed all general stopwords from the abstracts prior to the text-mining analysis, as well as the words that do not have any significant meaning in this particular case, such as model, validation, research, analysis etc. We also stemmize all the words, meaning that the words with the same root, for instance calibrate and calibration, are considered the same.

### Survey on model validation

To disclose individual views on the topics widely discussed in the validation literature, we employed a short online survey circulated among researchers and policy analysts in academia, policy organisations and industry. The survey contained three groups of questions about validation in general, and about scenario generation in particular. The first group of questions was about the organisational background, modelling experience, role and area of the respondents. For the organisational background and the modelling area, respondents could choose multiple options. The second group of questions contained a set of statements (Likert scale questions) reflecting the published viewpoints and issues, and asked to what extent the respondents agree or disagree with these statements This group also involved a question about the validation techniques used in the studies the respondents have been involved in. The last group of questions was about the validation of models specifically used for scenario generation. A set of statements were provided for Likert scale questions, and an additional question was asked about the prioritisation of scenario attributes important for a model’s validity.

For organisational background, the respondents were allowed to choose multiple options from Academic/Research Institute, Industry, Governmental policy organisation, Non-governmental policy organisation or None. Modelling experience asked for how many years the respondents have been involved in modelling or model-based studies. For the modelling role, the respondents were asked if they consider themselves model developers (with hands-on model building activities), model users (who use pre-existing models in research and analysis), with both of these roles, or none of these roles. For the modelling area, the respondents could select multiple options from a pool of research areas from energy to population dynamics.

The survey questions were prepared by the authors based on the literature discussed in the first two sections of this paper. The factors important in survey design, such as a common understanding, recalling the questions, and specifying what is to be rated and the continuum of rating^65^, were taken into account. To establish a common understanding, the survey included prior information about the modelling context, the definition of validation, philosophical roots, and the terms such as baseline scenario and scenario ensemble. Recalling a question with a different formulation is employed for the questions that require a comparison, such as representativeness vs. usefulness and the model output vs. structure. We used a continuum of 5 options on a Likert scale from Strongly Disagree to Strongly Agree.

In survey design, we unavoidably had to balance several trade-offs, especially because the modelling domain is very broad, and the views are very diverse. For instance, we intended to keep the survey as short as possible so that the respondents would not have to spend a long time. Due to this, we avoided long explanations and more questions. We also kept the questions general in order to suit to a wide variety of modelling backgrounds. We included a comments section, where the respondents could share their opinions in a more elaborate manner either on the subject or on the survey design. We assessed the survey questions, especially their understandability, in a pilot run among a small group of researchers, and revised them according to the feedback we received.

The survey was circulated among the professional networks of the authors via emails and social media, and the responses were collected over a two-month period. One hundred and eighty-eight responses were collected in total, and only 164 of them are included in the analysis since the rest did not answer the validation-related questions. Only 6 of the 188 respondents have mentioned in the comments that they had difficulty to understand some questions, and chose the neutral option as a response.

No personal data is collected in this survey, and all the responses are recorded anonymously. The respondents were informed about the potential use of their responses in scientific publications.

The entire list of questions can be seen at https://www.surveymonkey.com/r/IIASA_validation.

### Tests of independence

We investigate whether the responses to the Likert scale questions, hence the validation viewpoints, are dependent on the background of respondents recorded in the following three dimensions: Modelling role (developer, user, both, none), experience with modelling (less than 2 years, 2–5 years, 5–10 years, more than 10 years), and organisational background (academic, non-academic).

The statistical tests of independence we conduct are based on contingency tables for each question and the background factor. These tables are composed of the number of responses (observed frequencies) given to each Likert score. Table 2 exemplifies a contingency table, for Question 1 on general model validation and experience level.

**Table 2:** The contingency table for Question 1 and experience level

	Strongly agree	Agree	Neutral	Disagree	Strongly disagree
Less than 2 years	1	6	3	3	0
2-5 years	3	20	2	1	1
5-10 years	6	13	4	9	2
More than 10 years	15	34	10	10	4

Although the chi-squared test is usually used to test the independence of two variables of classification, Fisher’s Exact Test is suggested for cases when expected frequencies are less than five^66^, which is often the case in our data. Fisher’s Exact Test calculates the *p*-value for the null hypothesis of independence, based on a hypergeometric distribution of which the parameters are the observed frequencies in the contingency table. In particular, we use the software implementation of the Fisher Test in the R package stats^67^ (with Python interface) to calculate the *p*-values. For tables larger than 2 × 2, this calculation is computationally intense, which is why we use a Monte Carlo approximation with 10^8^ simulations. We reject the null hypothesis, and hence claim that the responses are significantly dependent on the background factor, for *p* < 0.05. Such *p*-values are not obtained for the questions on scenario generation. Therefore, we use *p* < 0.1 only to mention a potential relationship, not to claim dependence, as in the case of the effect of experience level on the responses to Question 1.

The resulting *p*-values are presented in the Supplementary Tables 3 and 4, and the statistically significant dependencies are visualised in Supplementary Figures 8 and 9.

### Code availability

This study uses custom computer code written in Python for the text-mining of academic publications, and to analyse the data collected from the survey. All the scripts (IPython Notebooks) can be accessed on https://github.com/sibeleker/Validation_Perspectives, or available from the corresponding author upon request.

## Supplementary information

Supplementary Information

Peer Review File

Description of Additional Supplementary Files

Supplementary Data 1

## Data Availability

The publication data used in this study is retrieved from the Scopus database originally, and available on https://github.com/sibeleker/Validation_Perspectives/tree/master/Topic_Modelling/InputDataset. The data collected through the online survey is anonymous and available online on https://github.com/sibeleker/Validation_Perspectives/tree/master/Survey. The data files are also available from the corresponding author upon request. The source data underlying Figs. 1–4, and Supplementary Figs 1–11 are provided as a Source Data file.
