# Supplementary material for: Practice and perspectives in the validation of resource management models
Source: Nat Commun. 2018 Dec 18;9:5359. doi: 10.1038/s41467-018-07811-9 (PMC6299083; doi:10.1038/s41467-018-07811-9)
Supplement: Supplementary file 1 — Supplementary Information [file 41467_2018_7811_MOESM1_ESM.pdf]

Eker et al. Supplementary information

Practice and perspectives in the validation of resource management models

Supplementary Figures

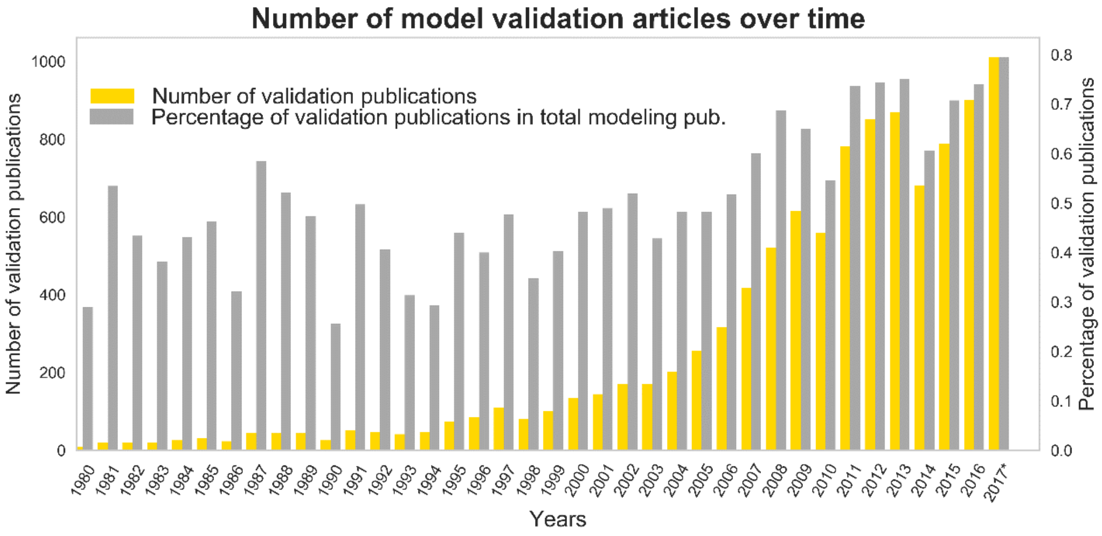

Supplementary Figure 1. Number of model validation publications over time and their percentage in the total modelling publications The text mining analysis is conducted on a collection of more than 10,000 academic publications retrieved from the Scopus database in the scientific disciplines specified in the Methods section. This figure shows that the number of model validation publications has significantly increased over time, yet it is still a small fraction of the total number of modelling publications. The increase in the number of publications has been substantial starting from the late 1990's, yet the percentage of validation-related publications has been less than 1 % throughout almost four decades. Therefore, it can be said that model validation receives more attention than in the past in terms of the absolute number of publications, yet only a small fraction of the modelling studies explicitly focus on validation regardless of time. Source data of this figure are provided in the Source Data file.

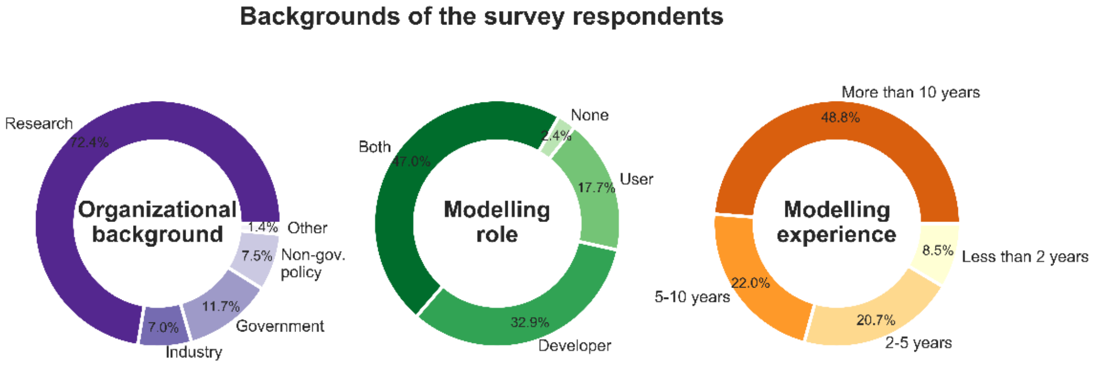

Supplementary Figure 2. Backgrounds of the survey respondents. The figure shows the backgrounds of the 164 survey respondents in terms of their organizational background, modelling role and modelling experience. A large majority is affiliated with universities and research institutes (72.4%; 95% if multiple affiliations are included), while the respondents from governmental or non-governmental policy organizations and industry constitute the remaining 25%. Regarding the modelling role, one third of the respondents are model developers, 17.7% consider themselves model users, and almost half of them report themselves as being both model developers and users. The respondents are considerably experienced in modelling, with almost half of them having more than 10 years of experience. Source data of this figure are provided in the Source Data file.

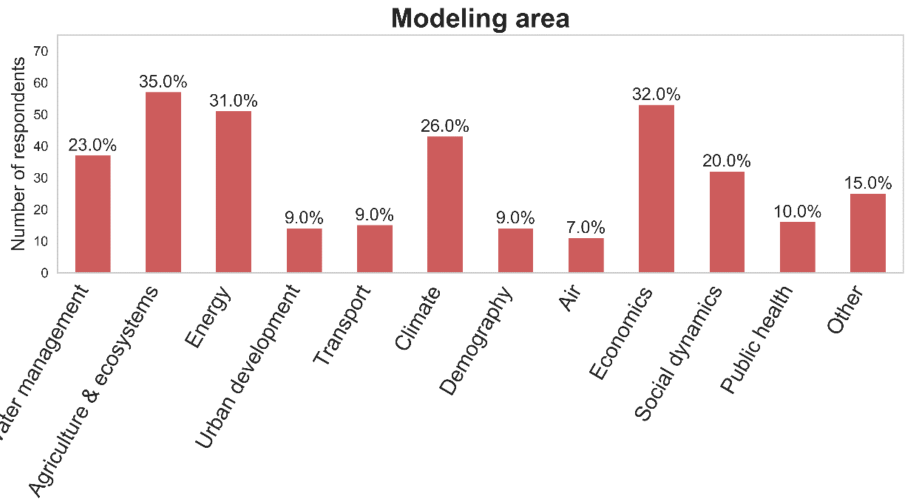

Supplementary Figure 3. Modelling areas the survey respondents have worked in. The figure shows the number and fraction of survey respondents who reported to have worked in each modeling area. Economics, climate, hydrology, agriculture and land use are the most common modelling areas the respondents have worked in, and most respondents have worked in multiple modelling areas. Source data of this figure are provided in the Source Data file.

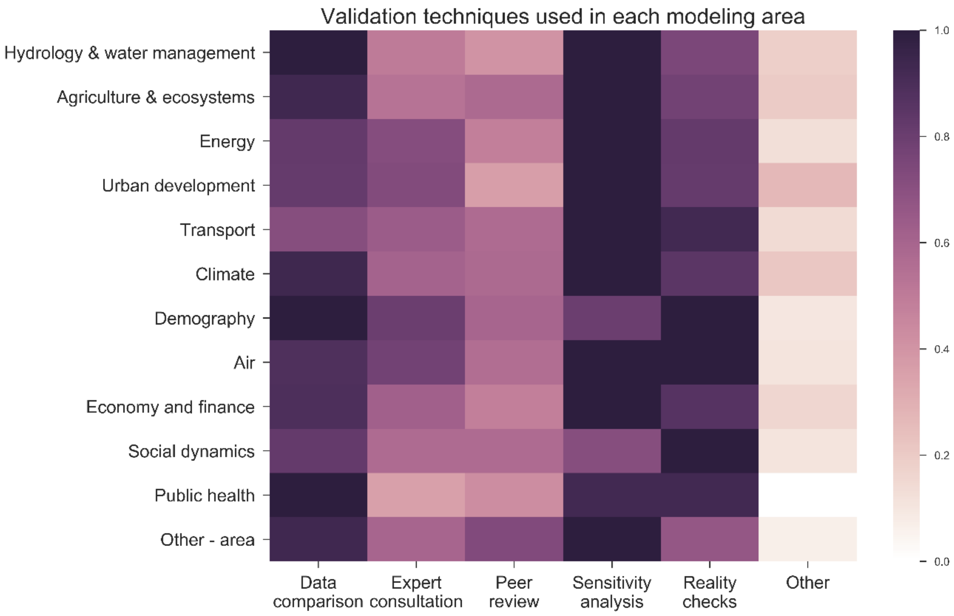

Supplementary Figure 4. Validation techniques used by the survey respondents in each modelling area. The figure visualizes the validation techniques used in each modelling area as reported by the respondents. The darker a cell in each row, the more commonly used the corresponding validation technique in this modelling area. Sensitivity analysis is the most commonly used technique in almost all areas, with the exception of demographics, social dynamics and public health. Comparison to historical data is another very common technique, yet it is outranked by sensitivity analysis, especially in areas where data is not expected to be rich due to recent development, such as transport, energy and urban development. Reality checks, in other words testing if the model demonstrates the expected behaviour under certain conditions, is another commonly used technique in all areas. As for the informal techniques such as peer reviews and expert consultations–they are rarely used compared to the formal techniques. Source data of this figure are provided in the Source Data file.

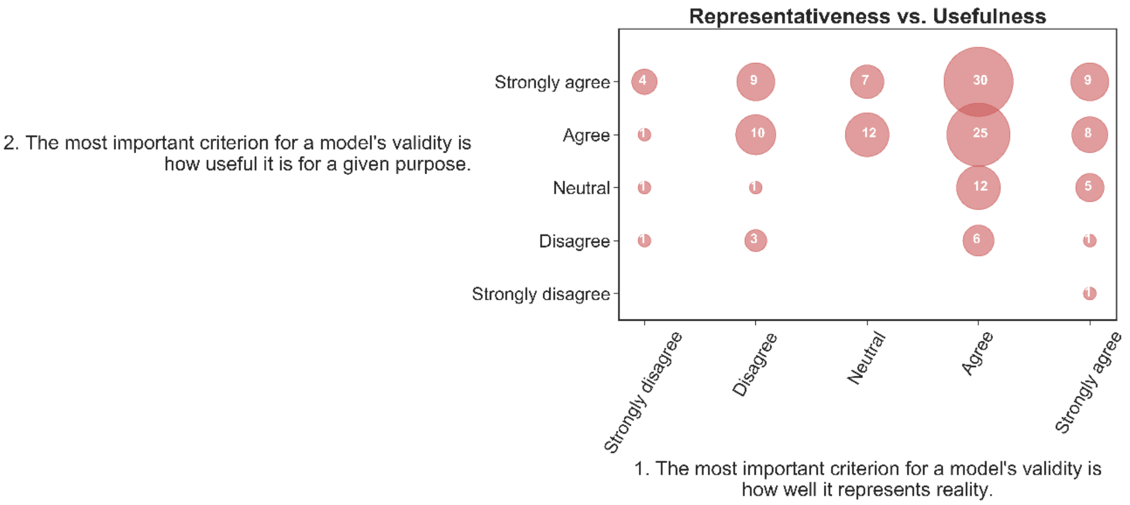

Supplementary Figure 5. Responses to the survey questions on model representativeness and usefulness. The first two Likert scale questions in the survey asked whether the most important validity criterion is representativeness (Question 1) or usefulness (Question 2). In total, 67% of the respondents agree or strongly agree with Question 1, while the 79% agree or strongly agree with Question 2. This figure illustrates how the responses given to these two questions coincide. The size of circles represent the number of responses given to Question 1 on the x-axis and the number of responses given to Question 2

on the y-axis. For instance, 30 respondents agree with Question 1 and strongly agree with Question 2. As seen in the figure, the majority of respondents who agree with one of these questions agree with the other, too. Therefore, this figure supports the conclusion that practitioners value both the usefulness and representativeness, and a dichotomy does not exist. Source data of this figure are provided in the Source Data file.

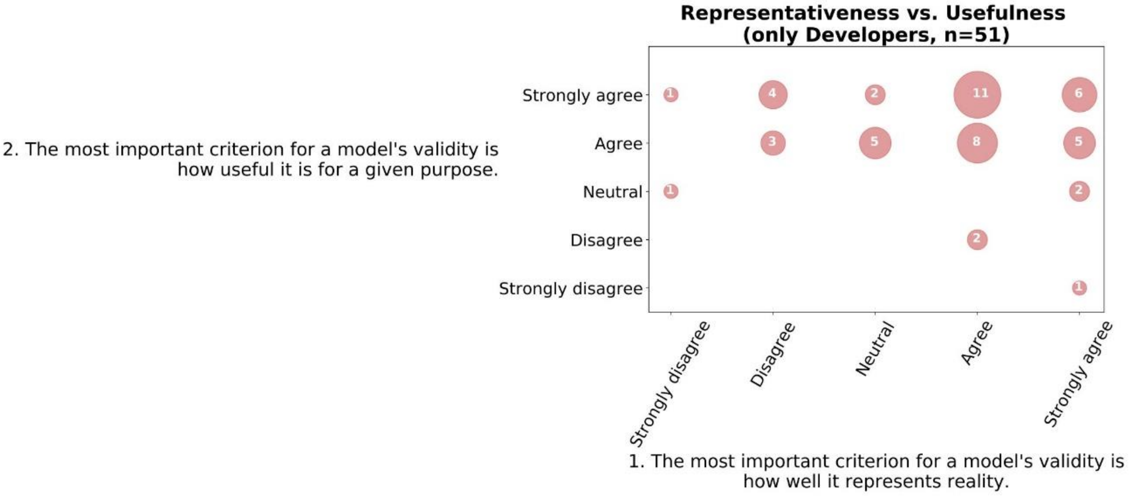

Supplementary Figure 6. Responses to the survey questions on model representativeness and usefulness only by model developers. The responses to Question 2 on usefulness are dependent on the modelling role. Model developers tend to agree with usefulness being the most important validity criterion more than model users. This figure visualizes the absolute number of responses given to this question on usefulness only by model developers, with respect to the question on representativeness. The majority of developers agree or strongly agree with the representativeness question. Very few developers remain neutral or disagree with Question 2 on usefulness, regardless of their responses to the representativeness question. In other words, 88% of model developers agree or strongly agree with usefulness being the most important criterion. Therefore, model developers tend to value usefulness more uniformly than they value representativeness. Source data of this figure are provided in the Source Data file.

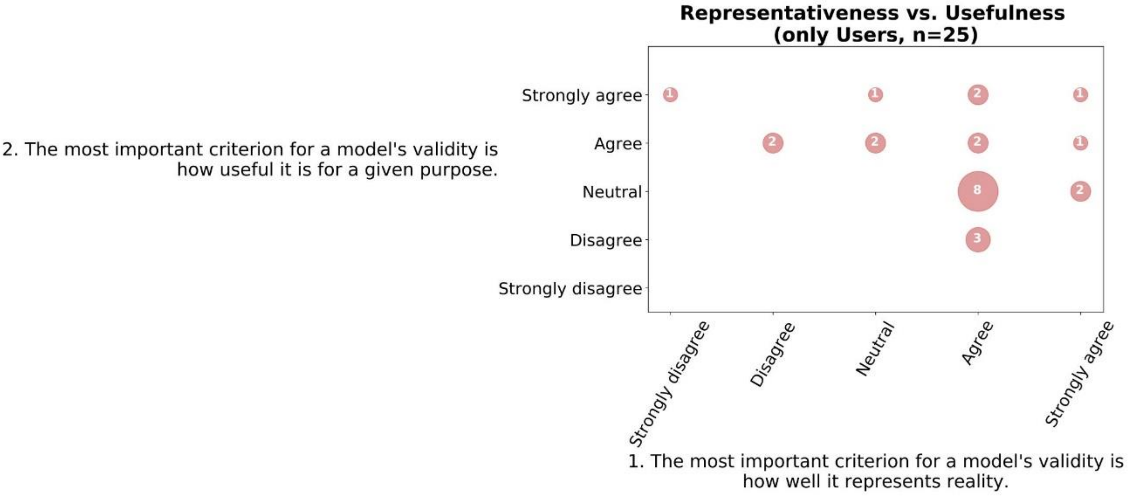

Supplementary Figure 7. Responses to the survey questions on model representativeness and usefulness only by model users. This figure visualizes the absolute number of responses given to the questions on representativeness and usefulness only by model users. Only 48% of users agree with the

statement on usefulness (Question 2), while the 52%, who favor representativeness, remain neutral or disagree. Therefore, a lower fraction of model users value usefulness compared to the model developers. The division between representativeness and usefulness is more apparent among the users. Source data of this figure are provided in the Source Data file.

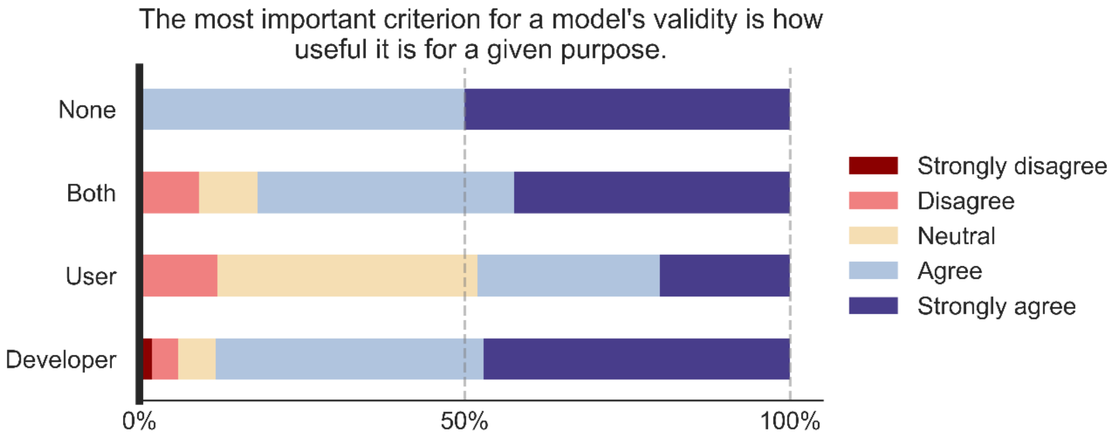

Supplementary Figure 8. Responses to Question 2 on model validation with respect to the modelling role. This figure illustrates the percentage of responses to Question 2, which is about usefulness as the most important validity criterion, for each modelling role. A higher fraction of model developers than users agree that the most important validity criterion is usefulness. Source data of this figure are provided in the Source Data file.

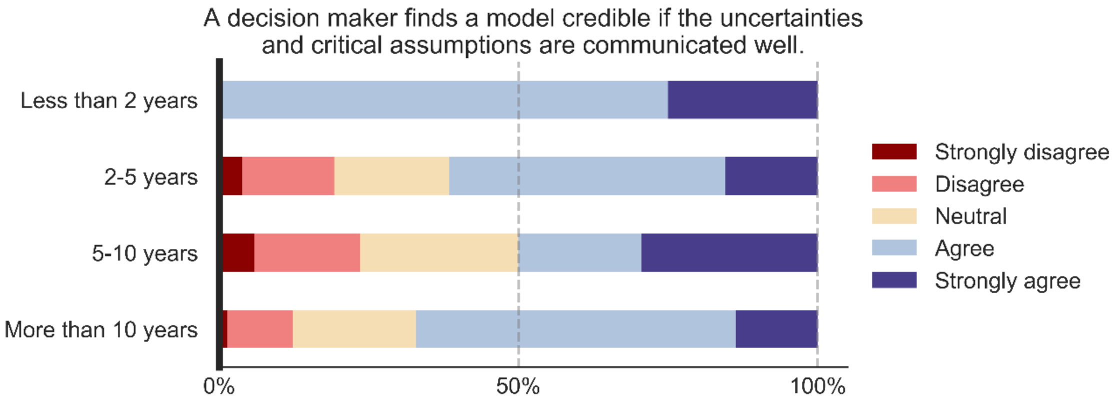

Supplementary Figure 9. Responses to Question 8 on model validation with respect to experience level. This figure illustrates the percentage of responses to Question 8, which is about the decision-makers' view on transparency, for each experience level. The respondents with moderate experience tend to disagree that decision makers expect clear communication of uncertainties and assumptions more than the ones with very high and low experience. In other words, more of the respondents with very short or very long experience acknowledge the decision-makers' demand for the communication of critical assumptions and uncertainties. Source data of this figure are provided in the Source Data file.

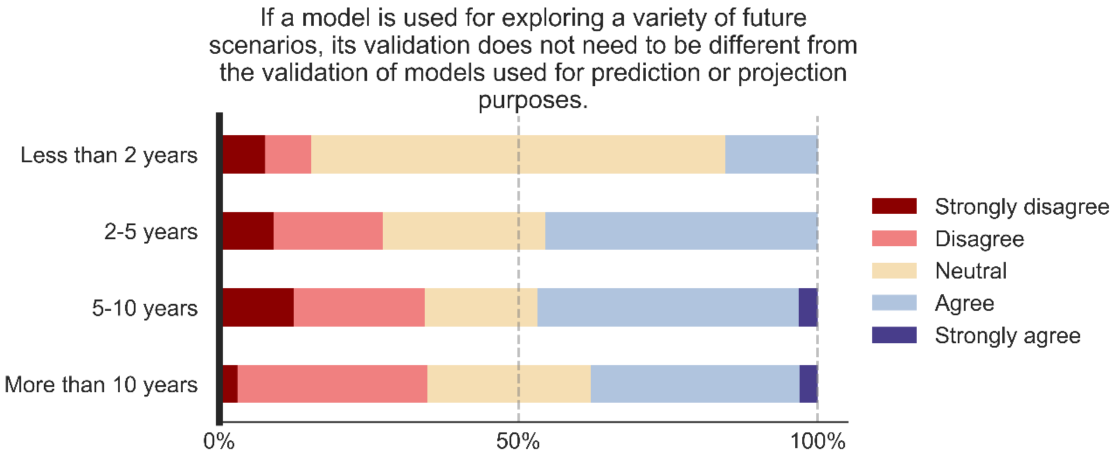

Supplementary Figure 10. Responses to Question 1 on model validation in the scenario generation context with respect to experience level. This figure shows the percentage of responses to Question 1 in the scenario generation context, about following a different validation approach, with respect to experience level. A higher percentage of the respondents with medium experience (2-10 years) agree that model validation should not be performed differently when the model purpose is scenario generation. Source data of this figure are provided in the Source Data file.

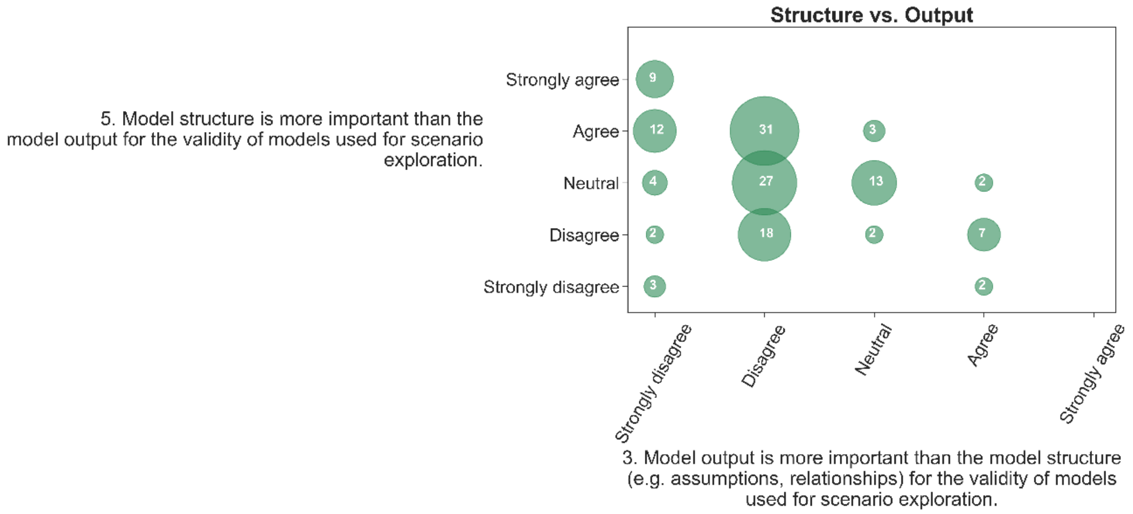

Supplementary Figure 11. Responses to the survey questions on model structure and output. The survey questions in the context of scenario exploration included two statements to compare the relative importance of model structure and output in validation. Question 3 stated that model output is more important than the structure, while Question 5 implied a higher importance of the structure. In total, 79% of respondents disagree with the relative importance of model output. This figure shows the number of relative responses given to these two questions. Among the respondents who disagree or strongly disagree with higher importance of the output, the ones who agree with the higher importance of the structure constitute the majority (31 people). Still, a large number of respondents who disagree with the higher importance of model output remain neutral or disagree also with the higher importance of the structure. Therefore, it can be said that the practitioners do not consider model output more important than the structure in the validation of models used for scenario exploration. However, they are equivocal about the structure being more important than the output. The respondents who disagree with the higher importance of the output yet remain neutral or disagree also with the higher importance of the structure may value these two characteristics almost equally, or may be indecisive about their answers. Source data of this figure are provided in the Source Data file.

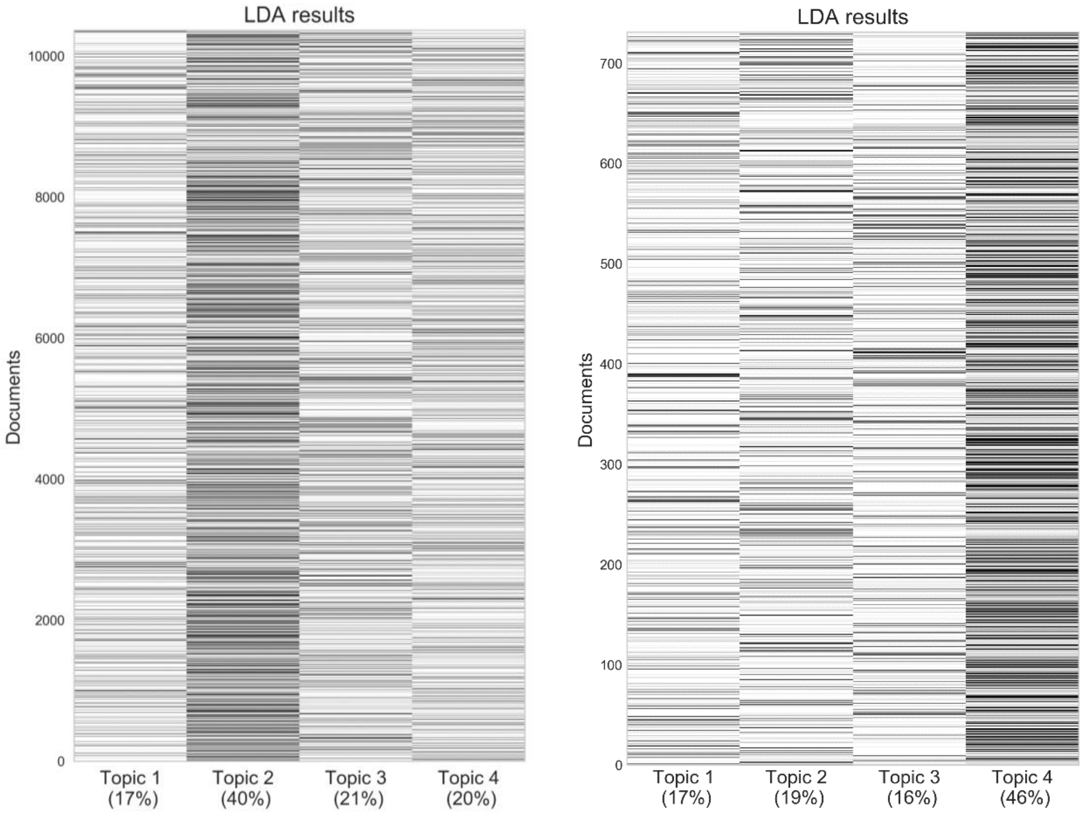

(a) (b)

Supplementary Figure 12. Document-topic pairs resulting from the LDA implementation for topic modelling (a) on the entire set of validation publications, (b) in the particular scenario context. The LDA algorithm used in this study to identify the main topics in the validation literature allocates each publication to a topic with a calculated probability. This figure visualizes these topic probabilities, where each line represents a document. The darker this line in the corresponding topics' segment (column), the higher the probability. Having heterogeneity across the columns in these figures indicate that the topics identified by the algorithm are distinct from each other. For instance, Topics 2 and 3 in (b) are the topics labelled as Hydrology and Climate Change and Ecosystems in the scenario-oriented validation publications. Most documents associated with Hydrology and Climate Change with high probabilities have a low association with the Ecosystems topic, and vice versa. This implies that most publications distinctively address these two topics, while the ones that integrate both are few.

Supplementary Tables

Supplementary Table 1. The word distributions of the four topics identified in Dataset I. This table presents the topic-word pairs visualized in word clouds in the main text. Namely, this table lists the top 50 words associated with each of the four topics identified in Dataset I on general model validation publications, and the point estimates of the word distribution in each topic. These point estimates represent the probability of a topic to include the corresponding word.

Rank	METHODS		EMISSIONS AND ENERGY		AGRICULTURE AND HYDROLOGY		ECOSYSTEMS	
1	data	0.0127	simul	0.0091	soil	0.0193	predict	0.0172
2	system	0.0108	concentr	0.0089	water	0.0158	speci	0.0135
3	evalu	0.0092	flow	0.0080	simul	0.0129	data	0.0125
4	test	0.0088	system	0.0076	data	0.0117	forest	0.0092
5	method	0.0085	measur	0.0074	predict	0.0103	variabl	0.0071
6	simul	0.0079	predict	0.0069	calibr	0.0092	estim	0.0070
7	develop	0.0074	data	0.0068	measur	0.0067	tree	0.0070
8	process	0.0071	experiment	0.0067	observ	0.0066	area	0.0065
9	base	0.0063	wind	0.0063	estim	0.0065	distribut	0.0065
10	propos	0.0062	develop	0.0061	crop	0.0064	popul	0.0060
11	applic	0.0050	condit	0.0059	paramet	0.0063	growth	0.0058
12	estim	0.0050	air	0.0055	climat	0.0057	habitat	0.0053
13	paramet	0.0049	energi	0.0055	hydrolog	0.0057	develop	0.0052
14	assess	0.0047	water	0.0053	area	0.0056	sampl	0.0050
15	inform	0.0046	test	0.0052	yield	0.0053	site	0.0047
16	network	0.0043	valid	0.0049	river	0.0045	spatial	0.0044
17	set	0.0042	temperatur	0.0048	field	0.0044	assess	0.0041
18	design	0.0041	process	0.0046	flow	0.0044	stand	0.0041
19	problem	0.0040	compar	0.0043	spatial	0.0044	evalu	0.0039
20	techniqu	0.0040	power	0.0043	evalu	0.0044	rang	0.0039
21	structur	0.0039	oper	0.0043	region	0.0043	method	0.0039
22	statist	0.0039	heat	0.0043	valu	0.0041	base	0.0038
23	provid	0.0038	numer	0.0041	manag	0.0040	ecolog	0.0037
24	time	0.0037	emiss	0.0039	increas	0.0039	regress	0.0037
25	comput	0.0037	method	0.0039	catchment	0.0038	manag	0.0036
26	predict	0.0037	rate	0.0039	runoff	0.0038	plant	0.0035
27	valid	0.0036	transport	0.0039	period	0.0037	test	0.0035
28	requir	0.0036	design	0.0039	variabl	0.0037	rate	0.0035
29	tool	0.0035	paramet	0.0039	time	0.0037	compar	0.0034
30	algorithm	0.0034	control	0.0036	compar	0.0037	measur	0.0033
31	case	0.0034	base	0.0036	error	0.0037	observ	0.0033
32	appli	0.0033	experi	0.0035	land	0.0037	effect	0.0033
33	import	0.0032	obtain	0.0034	develop	0.0037	level	0.0032
34	measur	0.0032	evalu	0.0033	condit	0.0036	potenti	0.0032
35	uncertainti	0.0032	calcul	0.0032	surfac	0.0036	environment	0.0031
36	complex	0.0031	investig	0.0030	product	0.0036	select	0.0031
37	dynam	0.0031	surfac	0.0030	sediment	0.0035	import	0.0030
38	framework	0.0031	time	0.0030	chang	0.0034	region	0.0029
39	select	0.0031	effect	0.0030	watersh	0.0034	individu	0.0028
40	effect	0.0030	effici	0.0030	temperatur	0.0033	densiti	0.0028
41	distribut	0.0030	sourc	0.0029	base	0.0033	biomass	0.0028
42	function	0.0029	pollut	0.0029	year	0.0033	fish	0.0028
43	discuss	0.0029	load	0.0029	assess	0.0032	age	0.0027
44	improv	0.0028	field	0.0029	scale	0.0031	relationship	0.0027
45	integr	0.0028	dynam	0.0029	process	0.0030	increas	0.0027
46	qualiti	0.0028	solut	0.0029	rainfal	0.0030	pattern	0.0027
47	implement	0.0028	increas	0.0029	input	0.0030	determin	0.0027
48	support	0.0027	equat	0.0029	total	0.0029	independ	0.0027
49	manag	0.0027	veloc	0.0028	site	0.0029	fit	0.0027
50	error	0.0027	plant	0.0028	season	0.0029	valid	0.0027

Supplementary Table 2. The word distributions of the four topics identified in Dataset II (on model validation and scenarios). One finding discussed in the main text is that uncertainty does not appear among the top words of the topics in Dataset II, which focused on scenario studies, hence was expected to involve an explicit uncertainty discussion. The table clarifies this finding, since uncertainty is among the most frequent words of only Hydrology and Climate Change topic, and in the 26th rank.

Rank	METHODS		HYDROLOGY AND CLIMATE CHANGE		AGRICULTURE AND HYDROLOGY		ECOSYSTEMS	
1	scenario	0.0146	climat	0.0233	water	0.0240	predict	0.0229
2	simul	0.0129	water	0.0174	soil	0.0211	speci	0.0141
3	system	0.0110	chang	0.0161	simul	0.0164	forest	0.0138
4	data	0.0109	scenario	0.0153	crop	0.0132	distribut	0.0095
5	develop	0.0098	increas	0.0151	concentr	0.0116	scenario	0.0090
6	test	0.0077	land	0.0148	manag	0.0111	data	0.0089
7	process	0.0075	impact	0.0130	yield	0.0109	climat	0.0084
8	evalu	0.0074	hydrolog	0.0103	predict	0.0105	popul	0.0083
9	base	0.0066	flow	0.0101	scenario	0.0086	variabl	0.0076
10	assess	0.0062	futur	0.0101	measur	0.0082	growth	0.0076
11	method	0.0051	river	0.0100	data	0.0075	chang	0.0070
12	design	0.0049	simul	0.0098	field	0.0071	estim	0.0068
13	time	0.0049	calibr	0.0095	groundwat	0.0070	tree	0.0067
14	requir	0.0047	watersh	0.0077	plant	0.0070	site	0.0061
15	tool	0.0046	load	0.0076	observ	0.0069	ecosystem	0.0058
16	applic	0.0046	period	0.0074	condit	0.0063	ecolog	0.0056
17	propos	0.0045	runoff	0.0070	agricultur	0.0062	region	0.0052
18	energi	0.0041	area	0.0067	evalu	0.0058	habitat	0.0050
19	inform	0.0041	sediment	0.0066	practic	0.0057	condit	0.0050
20	provid	0.0041	temperatur	0.0065	product	0.0055	area	0.0042
21	integr	0.0041	region	0.0064	nitrogen	0.0055	potenti	0.0042
22	case	0.0040	lake	0.0063	calibr	0.0052	manag	0.0041
23	dynam	0.0040	precipit	0.0062	area	0.0049	observ	0.0041
24	set	0.0039	catchment	0.0060	irrig	0.0049	rang	0.0041
25	valid	0.0038	spatial	0.0058	valu	0.0047	futur	0.0041
26	oper	0.0038	uncertainti	0.0057	loss	0.0046	suggest	0.0040
27	manag	0.0036	basin	0.0056	rate	0.0046	veget	0.0039
28	appli	0.0035	decreas	0.0055	treatment	0.0043	sensit	0.0039
29	work	0.0035	effect	0.0052	valid	0.0042	import	0.0039
30	decis	0.0033	data	0.0050	experi	0.0041	environment	0.0039
31	problem	0.0031	flood	0.0046	qualiti	0.0041	landscap	0.0038
32	complex	0.0031	discharg	0.0045	paramet	0.0038	compar	0.0037
33	optim	0.0031	variabl	0.0045	system	0.0038	respons	0.0037
34	environment	0.0030	annual	0.0045	test	0.0038	fish	0.0037
35	comput	0.0030	observ	0.0044	emiss	0.0035	rate	0.0037
36	order	0.0030	rainfal	0.0044	total	0.0034	conserv	0.0036
37	network	0.0029	project	0.0043	reduc	0.0033	stand	0.0036
38	support	0.0029	surfac	0.0043	growth	0.0033	stock	0.0036
39	specif	0.0029	base	0.0042	fertil	0.0033	paramet	0.0035
40	implement	0.0028	valid	0.0042	increas	0.0033	evalu	0.0034
41	compar	0.0028	urban	0.0041	region	0.0033	current	0.0033
42	exist	0.0028	reduc	0.0040	compar	0.0033	increas	0.0033
43	effect	0.0027	qualiti	0.0040	rang	0.0032	carbon	0.0033
44	import	0.0026	assess	0.0040	applic	0.0032	effect	0.0033
45	measur	0.0026	level	0.0039	conserv	0.0032	soc	0.0032
46	control	0.0025	stream	0.0035	estim	0.0031	biomass	0.0032
47	analys	0.0025	bv	0.0035	transport	0.0031	indic	0.0032
48	improv	0.0025	locat	0.0035	effect	0.0031	project	0.0032
49	estim	0.0025	season	0.0034	china	0.0031	error	0.0031
50	polici	0.0025	investig	0.0034	level	0.0030	measur	0.0030

Supplementary Table 3. p-values resulting from the tests of independence between the responses to the survey questions on general model validation and the background factors. The conclusions are based on the outcomes of the Fisher's Exact Test. We also conducted Chi-squared tests and the results were similar to those of the Fisher's test. Considering that a p-value smaller than 0.05 indicates a statistically significant dependence of responses to a factor, such dependence is found only between Question 2 and the modelling role, and Question 8 and the experience level. Source data of this table are provided in the Source Data file.

Question	Factor	p_Chi	p_Fisher
1. The most important criterion for a model's validity is how well it represents reality.	Role	0.620	0.547
	Experience	0.282	0.287
	Organization	0.676	0.791
2. The most important criterion for a model's validity is how useful it is for a given purpose.	**Role**	**0.013**	**0.014**
	Experience	0.950	0.953
	Organization	0.926	1.000
3. A match between the model output and historical data is a strong indicator that the model can provide accurate projections of the future.	Role	0.146	0.286
	Experience	0.301	0.139
	Organization	0.963	0.969
4. Multiple models can create the same output that matches with the historical data. Therefore, a model's validity cannot be linked to its replication of the past.	Role	0.347	0.438
	Experience	0.579	0.736
	Organization	0.836	0.758
5. Models cannot provide accurate projections; therefore, we cannot use them for prediction purposes.	Role	0.236	0.148
	Experience	0.270	0.322
	Organization	0.480	0.473
6. A model user, for instance a decision maker, finds a model credible if it can replicate the historical data.	Role	0.684	0.530
	Experience	0.939	0.944
	Organization	0.763	0.632
7. A decision maker finds a model credible if it is comprehensive and detailed.	Role	0.547	0.571
	Experience	0.176	0.155
	Organization	0.354	0.388
8. A decision maker finds a model credible if the uncertainties and critical assumptions are communicated well.	Role	0.293	0.325
	**Experience**	**0.086**	**0.039**
	Organization	0.202	0.193

Supplementary Table 4. p-values resulting from the tests of independence between the responses to the scenario generation questions and the background factors. None of the resulting *p*-values are smaller than 0.05; therefore a dependence conclusion cannot be derived. Only for Question 1 and the experience level, the *p*-value is smaller than 0.1, therefore we mention it as a potential significant factor. Source data of this table are provided in the Source Data file.

Question	Factor	p_Chi	p_Fisher
1. If a model is used for exploring a variety of future scenarios, its validation does not need to be different from the validation of models used for prediction or projection purposes.	Role	0.664	0.611
	**Experience**	**0.102**	**0.097**
	Organization	0.286	0.143
2. The validation of models used for scenario exploration should be based on their output in a baseline scenario.	Role	0.542	0.370
	Experience	0.228	0.118
	Organization	0.883	0.932
3. Model output is more important than the model structure (e.g., assumptions, relationships) for the validity of models used for scenario exploration.	Role	0.460	0.417
	Experience	0.271	0.261
	Organization	0.547	0.404
4. The validation of models used for scenario exploration should be based on the resulting scenario ensemble.	Role	0.781	0.751
	Experience	0.461	0.600
	Organization	0.523	0.536
5. Model structure is more important than the model output for the validity of models used for scenario exploration.	Role	0.967	0.979
	Experience	0.070	0.141
	Organization	0.871	1.000
