# Supplementary material for: Practice and perspectives in the validation of resource management models
Source: Nat Commun. 2018 Dec 18;9:5359. doi: 10.1038/s41467-018-07811-9 (PMC6299083; doi:10.1038/s41467-018-07811-9)
Supplement: Supplementary file 3 — Description of Additional Supplementary Files [file 41467_2018_7811_MOESM3_ESM.docx]

**Description of Additional Supplementary Files**

**File Name:** Supplementary Data 1

**Description:** This file includes the source data behind the figures used in the manuscript file. Each sheet of the file corresponds to the figure referred in the sheet name. In particular, Sheet 1 and 2 named Figure 1 and Figure 2, respectively, include the word frequencies of the top 50 words of each topic identified by the topic-modelling algorithm in the model validation publications. Sheet 3 and 4 named Figure 3 and Figure 4, respectively, show the number and fraction of responses given to each survey question on the Likert scale.
