# Supplementary material for: Practice and perspectives in the validation of resource management models
Source: Nat Commun. 2018 Dec 18;9:5359. doi: 10.1038/s41467-018-07811-9 (PMC6299083; doi:10.1038/s41467-018-07811-9)
Supplement: Supplementary file 2 — Peer Review File [file 41467_2018_7811_MOESM2_ESM.docx]

# **Reviewers’ Comments – Round 1**

**REVIEWER 1**

The authors present the results of a quantitative text-based literature survey, and a quantitative survey of practitioners, about model validation in the context of the use of models in environmental decision. Unfortunately the underlying concepts (e.g. positivism versus relativism) seem to be out of date, several of the questions in the survey appear to be poorly worded and ambiguous, and the paper is ultimately without any suggestion at all about what should constitute good practice in the field – it sends with the vague generalization that “scenario-specific aspects that relate to sampling and the analytical framework, such as plausibility, consistency and diversity, can be incorporated into the validation of such models.” (….just what does that mean? Would we knowingly include implausible or inconsistent models in any analysis? Though I agree that there are many studies with insufficient diversity i.e. not taking adequate account of epistemic uncertainties).

I am not sure that any practitioner, however skeptical of the ability of models to predict the future, would not agree that some sort of model validation (of process representations, boundary conditions, and other assumptions and auxiliary conditions) is required. In some situations this will necessarily qualitative because no empirical data are available (which includes all cases of predicting future scenarios – we cannot ensure that any model will produce a plausible future scenario as stated on p10, L8); in others it will be possible to compare model predictions against some historical data before making any future predictions. Both necessarily involve uncertainties, including epistemic uncertainties – that is what makes validation so problematic but as the authors note, their literature analysis did not reveal uncertainty as an important topic. It is therefore out of date. It is surely now accepted that an adequate validation strategy must allow for uncertainty in
process representations, parameters and boundary conditions. And it is rather easy to do so, for both qualitative and quantitative evaluation, within a Bayesian context. But, really rather surprisingly, Bayes or Bayesian is not mentioned anywhere in the paper. We do not have to be formal Bayesians - a formal likelihood Bayesian statistical context cannot easily deal with epistemic uncertainties – but in Bayes original formulation of updating the odds of a hypothesis given some evidence, some models (or ensembles of models) will be weighted more than others. Even though the authors suggest that in many situations “appropriate probability distributions cannot be elicited”, it is still necessary to make choices about what to run, so some possibilities will be given prior likelihoods of zero. We should also leave open the opportunity to refine that choice in evaluation and reject some of the models run (after taking account of relevant uncertainties of course).
Thus we can operate within a Bayesian framework, without being formally statistical. We do not have to be either positivist or purely relativist.

So the question then is to whether this survey paper is valuable. I have to conclude not in that it does not say anything about the state of the art or good practice but is rooted in the past.

Some additional comments.

1. P2 Section on model validation. There is no real difference between decision making and science. Both would want to use the best or fit-for-purpose prediction methodologies available and the comments about environmental modelling are also a form of usefulness for purpose.

2. P2 L36 I do not see many environmental modellers who can justify a positivist stance these days.

3. P2 L41. Example of ambiguous text: “participatory approaches to enhance the public reliability of models” - what does that mean?

4. P3 L2. Grammar of lists

5. P3 L8 “measurement of the data itself … subjective” - data should be plural; and how can the measurement be subjective? It might be uncertain, or subject to commensurability issues but what do you mean by a subjective measurement?

6. P4 L1. “yet do not elaborate on the sampling and logic of the connection between analysis and policy recommendations.” What does that mean?

7. P5 Fig1. Not clear if these topics are overlapping or mutually exclusive?

8. P5 L15. Decoupling. Is this a limitation of deciding on a number of topics in your analysis? Hydrology for example cannot be physically decoupled from crop models!!!

9. Fig3. Q4. Logically inconsistent. The problem of equifinality of model outputs does not imply that those models cannot be valid in predicting the future as any evaluation/validation has not (yet) rejected them.

10. P8 L27. “whether the models used for scenario generation should be validated based on a baseline scenario (Question 2) as in data-driven validation, or based on the ensemble of scenarios so that the extent to which the model captures uncertainties”. Not sure what you mean here. Clearly an ensemble of models can usually be found that are consistent or acceptable in some sense with past observations (and uncertainties in those observations). But we cannot evaluate an ensemble of scenarios of the future, except by some expert qualitative judgment (with all the problems of expert elicitation). But we also do not know the uncertainties in the future, so the model cannot be known to capture those uncertainties, except in so far as they are expert assessments of potential uncertainties. This needs more clarification about what you mean in terms of actual practice.

11. Figure 4 Q2. Also somewhat ambiguous – baseline scenario could be interpreted in a number of ways?

12. P10 L7. But there are already measures and frameworks for evaluating ensemble

**REVIEWER 2**

I noted about 30 comments on the pdf files. Indeed the authors are brave in attempting to chart such a vast and complex field. I agree with some of the conclusions, e.g. "Using models to explore multiple plausible futures instead of predicting a best-estimate future can still be considered a niche" and have some reservation for the broad categorizations used in the article (relativism, policy maker,...).

I would disagree that "a dichotomy does not exist among practitioners" - I believe that the field of modelling is extremely unruled, with each discipline going its own way. There are historical reasons and path dependenciens in the difference among disciplines.


It is a good conclusion that the science - policy (and technology) interface would deserve more study.

I added a few references. I notice that although the authors quote a work of Oreskes they do not report Oreskes' main point: that models can be used 'via negativa', e.g. to rule out possible options/inferences. I added in particular one more reference from Oreskes where the issue of validation is very well treated.

**REVIEWER 3**

Reviewer: Joseph Guillaume

The topic of validation is of substantial importance in many modelling-based fields and by providing a text analysis + survey, this article is likely to be highly cited as an indication of the status quo.
The article is also quite well written, and provides a nice overview of validation as a topic. I also personally appreciate how validation has been tied to more recent exploratory modelling work.
I'm usually a little sceptical of word clouds, and I still have nagging doubts as to whether a tabular presentation might give a more honest presentation, but the application of topic modelling seems to provide a nice contribution in this case.
There are some obvious issues with survey design (e.g. influence of wording of questions, size and representativity of sample are not discussed), but it already seems very useful, and worth publishing. I hope the (anonymous) data will be made available for further work.

There are, however, also quite a few conclusions that I feel are not entirely supported by the data, or where more nuanced interpretation might be needed. Discussion of methodological limitations would certainly be beneficial (at least in the supplement)

More generally, I feel the paper is a little vague in terms of its contribution. I am personally quite interested that it "investigates existing validation viewpoints" and expects "to provide model developers and users with reflections on and insights into their practice". I also agree that the results can in principle be used to "further elaborate on the requirements for a model’s validity and its contribution to decision-making." I would, however, not be surprised if less interested reviewers consider that the paper lacks a clear core message, and would be improved by providing some clear recommendations or paths forward from this work. The overall feeling is simply that data plays an important role (mentioned several times, but not actually that surprising to many people), and that there are diverse perspectives within the modelling community (not sure where the authors suggest we go from there).

I would therefore recommend publication, after the authors have had a chance to address issues raised.


Detailed comments
-----------------

p1 L21 "Some of these models have a normative stance and generate recommendations based on optimal decisions, while some others are descriptive and derive long-term projections that can help to assess the consequences of decision alternatives."
You talk about exploratory modelling later, but I think this dichotomy provides a false start to the topic.
Modelling can be descriptive in different ways. Consolidative vs exploratory modelling are both descriptive, but fundamentally different. Similarly, hard systems science vs soft systems science models have long distinguished between modelling that attempts to capture (objective) reality vs a (subjective) point of view.
Also, not all normative modelling frames its results as optimal decisions, only as "what should be done"
A possible solution: "Some of these models have a normative stance, generating recommendations for action. Others aid decision making in other ways, while still capturing the complexity ..."


p1 L36 "Validation is the modelling step employed to deal with such criticism and to establish a sound grounding for models at the science-policy interface."
Perhaps better to say "Validation is a modelling step commonly employed to ..."
At the moment, it feels like validation has been singled out at the expense of uncertainty analysis, sensitivity analysis, robustness assessment
(Edit: I note in the supplement that SA has been included in validation. This probably needs some clarification)
More broadly, I think at this point it would be useful to give a very high level definition of validation that distinguishes it from other techniques for dealing with model credibility in decision making.
p2 gets into definitions in more detail, but something is needed here already.
In my own work, I've identified 18 different ways of framing the role of uncertainty that are in common use to deal with criticism. Concepts related to validation only form a small (but commonly used) part of this.
Guillaume JHA, Helgeson C, Elsawah S, Jakeman AJ, Kummu M (2017) Toward Best Practice Framing of Uncertainty in Scientific Publications: A Review of Water Resources Research Abstracts. Water Resources Research, July. doi:10.1002/2017WR020609


P2 L4 "representation accuracy plays a less important role."
It would be useful to explain why, e.g. "because the representation of the system is expected to be uncertain, and that uncertainty is dealt with by other means in decision making"


p2 L5 "validation viewpoints on and approaches to general model validation practice"
Delete first "validation"?


p2 L6 "when models are used specifically for scenario generation to acknowledge uncertainty"
A little awkward
"when models are used specifically to generate scenarios used to explore uncertainty"?


p4 L12 "The first observation on this body of literature is that the number of model validation publications has significantly increased over time."
Is this simply because total number of publications has increased?
Is the number of model validation publications increasing faster than total number of publications?
Edit: Supplement 1 addresses this question, but shows large variation in % of validation-related publications. I would prefer to see a test that the increase is indeed statistically significant (it does look like it might be). I'm not sure I would call the increase substantial though, e.g. 1981, 1987, 1991 vs recent times.


p5 L15 "In both topics, uncertainty receives no attention,"
What do you mean by this? How would topic modelling have given it attention?
Would you expect the word stem "uncertain-" to come up?
I assume the word is present in the corpus (even if it is only abstracts). Would changing any decisions in the analysis cause the word to appear?
You might also want to comment/acknowledge factors that might lead to the word "uncertainty" not being used in the abstract
(I touch on this issue in broad terms in the paper cited above)


p7 L7 "Therefore, a dichotomy does not exist among practitioners"
I don't think this can be concluded from the results shown. You would have to show results of the two questions combined.
Perhaps the 20% who disagreed with Q1, fully agreed with Q2
Similarly 8% disagreeing with Q2 might fully agree with Q1.
I would hypothesise three groups: focussed on reality, usefulness, and both
The wording of the question is also ambiguous. Some respondents may have felt they were only allowed to choose one, while others may have felt permitted to say both are "the most important", given that the question is repeated.


p7 L13 "would be expected to favour usefulness more than representation accuracy."
However, this would be consistent with the notion that model users value models primarily for the insights gained as a result of their predictive or representation accuracy. i.e. if doesn't represent (something) well enough, then it's not going to be useful.
Other questions might clarify whether this is the case?


p7 L24 "objections to using data oriented validation approaches due to the impossibility of a purely positivist validation are not widely acknowledged by practitioners."
I don't think this statement is supported. It is quite possible for practitioners to acknowledge the objections but dismiss them as irrelevant to their context.
I believe the paradox can easily be resolved from a sense-making perspective. Practitioners acknowledge that purely positivist validation is impossible, but within their work, it is still useful to act as if positivist validation were possible - it helps to make sense of what results to trust for a given purpose, and to convince others that one's research is worth paying attention to. Investment firms have to include the disclaimer that past results are not predictive of future performance precisely because they *can* be, and they are still *used* that way.
Personally, I'm a pragmatic relativist - I find it very useful to appeal to positivist arguments in some circumstances.
I would be happy if this sentence was rephrased to say something like "objections to using data oriented validation approaches due to the impossibility of a purely positivist validation do not result in practitioners completely rejecting those approaches"


p8 L36 "it can be said that the respondents favour a structure focused validation when the model purpose is scenario generation"
It seems Q5 is the converse of Q3 (and it's nice to see this kind of check). However, the 79% that think model output importance <= model structure importance only translates to 40% who think that model structure importance => model output importance.
Presumably the remaining 39% think they are equally important or are unsure, or were influenced by question wording. The point is not quite as unequivocal as this sentence makes it seem - a few extra words of nuance are needed.


p9 "Many scholars have argued that data oriented validation is not sufficient, and that validation should include conceptual aspects and participatory approaches to enhance the public reliability of models. Our results show that this view has not been widely implemented,"
I don't think this point is sufficiently supported. You would need a specific analysis of whether conceptual aspects and participatory approaches are being used *in addition to* data-based methods.
I would be happy with a wording in the direction of "Our results do not indicate wide implementation of this view" (i.e. you have absence of evidence, not yet evidence of absence)
Edit: Figure S.5 clearly shows that using both data comparison and sensitivity analysis/reality checks is common. That seems to contradict the conclusion here.


p9 L14 "relative difficulty of defining usefulness compared to representativeness,"
I would say "relative difficulty of measuring ..."
Defining representativeness is very difficult too, if you look deep enough into it.
However, it is relatively easy to come up with many widely transferable indicators (e.g. see Bennett et al.)
In contrast, usefulness is always obviously context-dependent, so any attempt to adopt a transferable indicator is likely to be controversial.
The effort in measurement comes from having to understand the application context in sufficient depth (see e.g. Haasnoot et al.) for the measurement to be credible. The following sentence about resources needed already seems to imply this.


p9 L21 "Yet, model structure is considered more important than the output in this context, as indicated by both the literature and practitioners"
See comment above. I don't think this statement is 100% certain.


p9 L18 "since the academic literature vastly emphasizes prediction, and most practitioners think that models can be used for prediction purposes"
I don't think this argument holds. As mentioned here and in Maier et al., an approach with multiple plausible futures is usually used in combination with a best-estimate approach. Different uncertainties in the model are dealt with in different ways. Therefore emphasis on prediction (in some form) will always exist.
I do still agree that "multiple plausible futures" is a niche, but not based on any evidence presented here.


p13 L3 "We reject the null hypothesis, and hence claim that the responses are significantly dependent on the background factor, for p<0.05. Such p values are not obtained for the questions on scenario generation, therefore we use p<0.1 to reject the null hypothesis of independence for these questions."
This sounds a little suspicious. It might be good to give a different justification, or to explain the impact of this decision on results.
Also revise Supplement p6 L2


Figure S.8 "A higher percentage of the experienced respondents disagree that model validation should be performed differently when the model purpose is scenario generation."
It seems to me that this is more a case of more experience=more confidence/stronger opinion. The results are still very divided.

# **Response to Reviewers – Round 1**

## Reviewer 1

We are thankful for your careful and critical review on our manuscript. Below we respond to your general comments and hope to clarify the objective and the arguments of the manuscript. We then summarize the revisions in the manuscript in a table format for the specific comments you have made.

You have stated “***the underlying concepts (e.g. positivism versus relativism) seem to be out of date***”. Thanks for raising this concern. The aim of the corresponding paragraph in the manuscript is to provide a brief overview of the philosophical roots yet not to discuss them in detail. According to the highly acknowledged publications on the philosophical background of validation (we refer to these in the text), these two are the underlying philosophies of science, both conceptually and historically. Although this division is not very strict currently, as our results show, positivism and relativism are still the underlying concepts. We think that time would not change the root of a temporal change. Regarding these philosophies of science, we considered constructivism instead of relativism as suggested by Reviewer 2. However, we found that there is no literature established on the role of constructivism in validation. Therefore, we keep these two as the underlying philosophies of science, and refer the reader to the sources we cite for further interest.

You have stated that ***several survey questions appear to be poorly worded and ambiguous***. We acknowledge the difficulty of designing such a survey that should appeal to a diverse audience from different modelling backgrounds. In the Methods section of the revised manuscript (P14 L14 – P15 L4), we have more extensively discussed the survey design, the reasons for the choices we made, and the comments made by respondents on the wording.

You mentioned that ***the paper is without any suggestion about what should constitute good practice in the field***. We understand the demand for suggestions about what constitutes a good practice. As we specify in the Introduction section of the original manuscript (P2 L6-13), the objective of this paper is to investigate the existing perspectives, i.e. the status-quo. We do not aim at proposing a good practice; therefore, do not elaborate on it. We hope that you can evaluate the manuscript with respect to this objective. Additionally, there are several good papers we refer which directly suggest good practice, for instance Ref. 29, 31, 32, 35. A related revision in the manuscript is in the Discussion section, where we formulate the implications of, and suggestions based on, the findings.

Regarding our text “scenario-specific aspects that relate to sampling and the analytical framework, such as plausibility, consistency and diversity, can be incorporated into the validation of such models.”, you have asked “...***just what does that mean? Would we knowingly include implausible or inconsistent models in any analysis? Though I agree that there are many studies with insufficient diversity i.e. not taking adequate account of epistemic uncertainties***”. We are glad to hear your agreement that diversity is insufficient in many studies. With this statement, we refer to a validation practice based on scenarios (a scenario ensemble) rather than a baseline scenario, if a model is used for scenario generation. Plausibility, diversity and consistency are scenario attributes, which are often used in evaluating scenarios. Therefore, we claim that these attributes used in evaluating scenarios can be brought into the validation practice, and can be used to evaluate the models that generate those scenarios, too. We have revised the text (P11 L43 – P12 L4) to clarify that these are desired scenario characteristics, which can be considered when model validation focuses on scenarios.

You have stated “***I am not sure that any practitioner, however skeptical of the ability of models to predict the future, would not agree that some sort of model validation … is required****.”* We are unfortunately not clear about which part of the manuscript you refer here, since we don't claim anywhere in the manuscript that practitioners object to the requirement for validation. It is true that validation is often not explicit. However, we do not think that this is because practitioners think that it is unnecessary.

You have mentioned that “***both [model predictions and data] necessarily involve uncertainties, including epistemic uncertainties – that is what makes validation so problematic but as the authors note, their literature analysis did not reveal uncertainty as an important topic. It is therefore out of date. It is surely now accepted that an adequate validation strategy must allow for uncertainty in process representations, parameters and boundary conditions****.*” We totally agree that uncertainty is one of the most important concepts in model validation, and an adequate validation strategy should allow for uncertainty in representations, parameters and model boundaries. Our text-mining results not showing the word "uncertainty" is an intriguing and counter-intuitive finding, indeed, as we discuss. We are hesitant to accept the claim that this finding is out of date, since it relies on a large set of publications, most of which are recent (Please see the Methods and Supplementary Information). In the revised manuscript, we discuss the potential reasons for this finding with respect to a recent publication on uncertainty framing.

Regarding your comment about the absence of a discussion on **Bayesian approaches**, we agree that some models or ensembles of models will be weighted more than the others in terms of plausibility. Still, we would like to clarify that we refer to “scenario ensembles” in this text, not “model ensembles”. We agree that the Bayesian approaches are commonly used to deal with uncertainty. In this manuscript, however, we focus on an alternative paradigm named as “multiple plausible futures”, which is used for situations where probabilities cannot be derived (P3 L32-34). Therefore, also considering the space limitations, we refrain from discussion the Bayesian approaches to deal with uncertainties.

You *conclude not in that the manuscript does not say anything about the state of the art or good practice, but it is rooted in the past*. On the one hand, we are glad to hear that our manuscript is well connected to the established past literature. On the other hand, we understand why our manuscript might have been found rooted in the past instead of the present. We kindly would like to remind that our findings are based on a publication dataset dominated by recent publications (P4 L24-25), and a very recent survey circulated about current practitioners. Therefore, for the subjects and issues that may seem out-of-date, we would like to clarify that they are not our claims, they are presently discussed in the literature and by practitioners. (The dichotomy between representativeness and usefulness can be considered an exception, and we are glad to show that it currently does not exist.)

| **Place in the Original Manuscript** | **Comment** | **Response** |
| --- | --- | --- |
| P2 L36 | I do not see many environmental modellers who can justify a positivist stance these days. | We are glad that you reflect on this subject from your personal experience. It is also our experience that many environmental modellers acknowledge that validation goes beyond a positivist stance. We state the presence of this view in the literature in the following sentences. ("Still, many studies suggest integrated frameworks that assess conceptual and methodological validity along with data and behaviour validity^31, 32, 33^, and some studies offer qualitative frameworks and participatory approaches to enhance the public reliability of models^5, 34, 35, 36^.") Still, we should also note that many of our findings both from text-analysis and the survey support the claim in P2 L36. |
| P2 L41 | Example of ambiguous text: “participatory approaches to enhance the public reliability of models” - what does that mean? | We have revised the text (P3 L1-2) to clarify that qualitative frameworks and participatory approaches are offered to involve stakeholder views in modelling studies, so that the public trust in such studies can be enhanced. |
| P2, L14-18 | There is no real difference between decision making and science. Both would want to use the best or fit-for-purpose prediction methodologies available and the comments about environmental modelling are also a form of usefulness for purpose. | Thanks for the comment. We would like to remind that this statement was about environmental modelling and decision sciences. About the difference between these two disciplines, we are following the papers we are citing. We agree that both decision-making and decision sciences would want to use a model that fits the purpose, relating to usefulness. In the revised manuscript, we have a more extended discussion of usefulness (P3 L13-23). |
| P3 L2 | Grammar of lists. | We have revised the paragraph to list the reasons for impossibility of a purely positivist validation in a grammatically correct and more understandable way. (P3 L3-11) |
| P3 L8 | “measurement of the data itself … subjective” - data should be plural; and how can the measurement be subjective? It might be uncertain, or subject to commensurability issues but what do you mean by a subjective measurement? | About the plurality of 'data', we would like to keep the current format, since we use it as 'information', not necessarily as the plural of 'datum'. According to the Merriam Webster dictionary, in such cases 'data' is being referred to by a singular pronoun. About the 'subjectivity', we agree that the word "subjective" is confusing. In the revised manuscript, we instead use "inference-laden" (P3 L9-11), since this better explains the argument in Oreskes et. al. (1994) (Ref. 23) that "What we call data are inference-laden signifiers of natural phenomena to which we have incomplete access" and "Data are never complete, inferences are always required, and we can never be certain which inferences are good and which ones are not as good." |
| P4 L1 | “yet do not elaborate on the sampling and logic of the connection between analysis and policy recommendations.” What does that mean? | In the beginning of this paragraph, three points are listed to focus on in the validation of exploratory models. The sampling strategy and the logic of connection between analysis and policy recommendations referred to the second and the third of these points, respectively. To clarify, we have revised the text and used the exact same wording so that the reader can relate it to the beginning of the paragraph (P4 L10-13). |
| P5 Fig1. | Not clear if these topics are overlapping or mutually exclusive? | The topics are indeed not mutually exclusive. To clarify this, we have revised the text in the Methods section (P13 L9-20), and added a statement about similarities in the caption of this figure, as well as a reference to the Methods section. |
| P5 L15 | Decoupling. Is this a limitation of deciding on a number of topics in your analysis? Hydrology for example cannot be physically decoupled from crop models!!! | We agree that most hydrology models cannot be decoupled from crop models. This is reflected on the results of text mining (Fig. 1), since the words like *crop* and *soil* are most defining alongside words like *water* and *hydrology*. To clarify this, we have added two sentences to the Methods section (P13 L14-16) about naming. Regarding the predefined number of topics, we agree that an algorithm that can distinguish between any number of topics in a dataset would have been ideal, but it does not exist to our knowledge. Therefore, we used LDA, the main topic modelling algorithm, which requires specifying the number of topics beforehand. |
| Fig3 Q4 | Logically inconsistent. The problem of equifinality of model outputs does not imply that those models cannot be valid in predicting the future as any evaluation/validation has not (yet) rejected them. | Thanks for raising this concern. We agree that equifinality does not imply that a model is not valid. It only implies that multiple models can replicate the past data; therefore, a model's validity cannot be solely linked to replication of the past. The survey question is prepared on this line, to mean that validity cannot be linked to the replication of the past due to equifinality. Therefore, we think that there is no inconsistency in this statement. We also would like to remind that the survey questions are not claims we are making, but statements we have asked to practitioners to understand their view. |
| P8 L27 | “whether the models used for scenario generation should be validated based on a baseline scenario (Question 2) as in data-driven validation, or based on the ensemble of scenarios so that the extent to which the model captures uncertainties”. Not sure what you mean here. Clearly an ensemble of models can usually be found that are consistent or acceptable in some sense with past observations (and uncertainties in those observations). But we cannot evaluate an ensemble of scenarios of the future, except by some expert qualitative judgment (with all the problems of expert elicitation). But we also do not know the uncertainties in the future, so the model cannot be known to capture those uncertainties, except in so far as they are expert assessments of potential uncertainties. This needs more clarification about what you mean in terms of actual practice. | We agree that the model cannot be known to capture those uncertainties, since we don't know what these uncertainties are. In this case, evaluating the scenarios with experts is surely a useful technique. In future research, we aim to investigate techniques the modelers can apply themselves prior to, or when there is no chance, to involve experts. What we mean in this manuscript is one-step back, on the conceptual basis of a validation approach. As you know, existing validation approaches center around a baseline simulation, whether it is to compare this simulation to the historical data, or to check its sensitivity to different input values and assumptions. What we mean is that, when a model is used to generate a scenario ensemble, should we consider this ensemble in validation, rather than the baseline simulation? To avoid confusion, we removed the text on capturing uncertainties, and we clarified that the survey informed respondents about these two terms. |
| Fig4 Q2 | Also somewhat ambiguous – baseline scenario could be interpreted in a number of ways? | The survey included a brief definition of "baseline scenario" and "scenario ensemble" before the questions. Therefore, the respondents were informed about the definition we use. This disclaimer in the survey, which can also be accessed through the link we provide in the Methods section, was as follows: "In the statements below, the 'baseline scenario' refers to a reference scenario obtained from the model, often based on the best available knowledge. The 'scenario ensemble' refers to a relatively large number of scenarios obtained from the model with alternative realizations of uncertainties." |
| P10 L7 | But there are already measures and frameworks for evaluating ensemble | We would like to clarify that this sentence refers to evaluating models that are used to generate scenario ensembles with the "multiple plausible futures" paradigm. We do not refer to evaluating model ensembles. To our knowledge, there are no validation frameworks for such models, and we will be glad to receive references if there are any. We clarified what is meant by a scenario ensemble in P3 L35-38. |

## Reviewer 2

We are thankful for your careful review on our manuscript and reflecting on our findings with personal expertise and experience. We are glad to hear that you agree with our conclusion that the science - policy interface (in the context of modelling) would deserve more study. We appreciate that you have suggested additional references to support our arguments. We have inserted their main points into our text accordingly, as described in the responses to the detailed comments below.

Regarding your disagreement that "a dichotomy does not exist among practitioners", we agree with your remark that modelling and validation practices differ among different disciplines, and different disciplines are not connected to each other. About this particular statement on dichotomy, we would like to remind that it refers to the dichotomy between the representativeness and usefulness view, which is not supported by the survey results we presented in this paper. We have revised the main text (P7 L15-17), also added supplementary material to clarify this survey finding.

| **Place in the Original Manuscript** | **Comment** | **Response** |
| --- | --- | --- |
| Abstract | Usefullness? | In the revised manuscript, we discuss the definition of usefulness more explicitly, following other comments and suggestions (P3 L13-23). We admit that this definition would have been useful already in the abstract. However, due to space limitations, there is no chance to discuss this in detail in the abstract. We hope that it becomes clear to readers in the main text. |
| P1 L21-23 | All models embed norms. … This categorization of models into normative and descriptive does not hold. | We agree that all models embed norms. Here, the purpose was to distinguish between two broad categories of optimal control and dynamic simulation models, which provide temporal predictions. We understand that this leads to confusion, as also stated by Reviewer 3. We have revised the text as suggested (P1 L21-25). |
| P2 L4 | This reference coulf be added here  Ravetz, J. R. (1997). Integrated Environmental Assessment Forum, developing guidelines for “good practice”, Project ULYSSES. Retrieved from http://www.jvds.nl/ulysses/eWP97-1.pdf | Thanks for the suggestion. We are familiar with the Post-Normal Science literature, and the quality assurance framework based on it. After reading the working paper you have suggested, since it is focus on "good practice" of model-based policy analysis rather than using models to explore a wide range of scenarios, we decided to add it as a validation approach that takes conceptual and methodological validity into account, as well as involving participatory approaches (P3 L1-5). |
| P2 L18 | I am not sure that Oreskes would subscribe to this sentence. (P2 L18) | We agree that this statement wouldn't be the view of Oreskes herself. However, we used this reference, because she refers to the general view and argues against it in that publication. Still, to avoid confusion, we now use another reference, Beven 2002 (Ref. 24) in which such a definition of validation in environmental modelling is more explicit. |
| P2 L33 | I subscribe to the need for synthesis but the envelop of relativism is really too broad and would be resisted by any scholar of science and technology or of history and philosophy of science.  Perhaps a broad categorization which would stand is that between natural and social sciences - with natural sciences tending to positivism and social sciences tending to constructivism. | Thanks for raising this concern. We understand that relativism sounds very broad for the model validation context, and constructivism indeed underlies the integrated validation approaches. Still, there is no literature developed on the constructivist view in validation. We could find this neither in the old publications that are highly acknowledged for the philosophical background of validation (we refer to these in the text), nor in a recent literature search. Therefore, since the aim of this manuscript is to provide a brief overview of the philosophical roots yet not to discuss them in detail, we refrain from extending this paragraph. The interested readers are referred to the three publications we cite, which discuss model validation with respect to different philosophies of science very fruitfully. |
| P3 L8 | A suggested reference here is Oreskes, N. (2000). Why Predict? Historical Perspectives on Prediction in Earth Science. In Prediction: Science, Decision Making, and the Future of Nature (pp. 23–40). | Thanks for recommending this very good chapter on the history of philosophy of science, the meaning of 'prediction' and the rise of demand for it in public policy and environmental research. We have added it as a reference in the corresponding line to support the argument on the impossibility of purely positivist prediction. |
| P4 L19-33 | Some courageous inference here. | Thanks for the comment. We understand that you are not in total agreement with our interpretation of the text-mining results. We would be thankful if you have given more explanation about what you disagree. We would like to note that, in the supplementary material, we have added the word frequencies underlying the word clouds that visualize the topics identified in the literature. We hope that it will clarify the conclusions we derive from these findings. |
| P5 L6-19 | I subscribe to the fact that hydrologists have been historically among the first to realize the impossibility of model verification, e.g. Hornberger, G. M., & Spear, R. C. (1981). An approach to the preliminary analysis of environmental systems. Journal of Environmental Management, 12(1).  and that system ecologists working e.g. in the tradition of Rosen do things differently. | We are glad to hear that our empirical finding is supported by your experience and the literature. |
| P7 L14 | I feel uneasy with the concept of usefulness. Also here it seems to me be too broad a categorization, but I understand the need for synthesis. Anyhow I suggest this reading:   Clark, W. C., & Majone, G. (1985). The Critical Appraisal of Scientific Inquiries with Policy Implications. Science, Technology, & Human Values, 10(3), 6–19. https://doi.org/10.1177/016224398501000302 | Thanks for the understanding the need for synthesis and the reference to Majone. The reason for not discussing a usefulness definition in detail was space limitations. We tried to overcome this by revising the text, emphasizing alternative model uses and incorporating Clark and Majone's views on the critique of scientific inquiry in policy contexts (P3 L13-23). Also, following Reviewer 3's suggestion, we clarified in P7 L23 (P7 L14 in the original manuscript) that model users are expected to favor usefulness unless their purpose is an accurate representation. |
| P7 L21 | This would imply that there is a problem, after all. | In the revised manuscript, we have emphasized this absence of consensus on equfinality principle in validation. |
| P7 L22 | I would disagree with question 5 myself. It is too draconian. | Thanks for stating your opinion. This statement was intentionally worded so, to represent the views at the ends of the spectrum. |
| P8 L2 | Agree. I believe that modelling communities operate in disciplinary syloses, e.g. epidemiologists don't mix with hydrologists and even less with behavioural psychologists. | We are glad to hear that our finding is supported by your personal experience and view on the modelling communities. |
| P8 L7 | It is crude to partition betwen practitioners and decision makers. Many l live in hybrid configurations where they are a mix of both. | We agree that practitioners and decision-makers are often not distinct groups. Similarly, most of our survey respondents have double roles as model users and developers. Still, we have this distinction in the survey questions in order to refer to people, for instance clients or public authorities, who actually use modelling results in their decision-making. We added "clients" as an example of 'decision-makers' in the revised manuscript. |
| P8 L17 | Interesting finding. There is a normative issue here concerning the analyst herself. Much depends on the expectation of what is needed (or what one can get away with). If the positivist view has traction and earn a salary why should I make my life more miserable by messing with uncertainties? | Thanks for the interpretation of this finding. We agree that such personal concerns play a role. The reason to mess with uncertainties, in that case, could be the demand by decision-makers, so to speak clients, who expect both a positivist validation and a clear uncertainty communication. We have inserted a sentence to mention that employment conditions can play a role in this view, too. |
| P8 L37 | I am appreciate the difficulty of the task yet I would be at loss to answer myself. Sensitivity analysis should be used to investigate if the results depends more upon the calibration data or upon the the calibration procedure.   What is a scenario ensemble and how was it arrived at? | Thanks for understanding the difficulty of this task within the word limits. Sensitivity analysis is indeed a commonly used validation technique, as our survey respondents reported, too (Supplementary information). However, in the context of scenario exploration, where this structure vs. output discussion belongs, using sensitivity analysis for validation is problematic. Sensitivity analysis aims to make models robust to input changes, and in the context of scenario generation, this can lead to reducing the model's ability to generate diverse scenarios. We inserted this to the revised manuscript (P4 L8-10) where we talk about the validation of exploratory models.  P3 L35-38 explains how scenario ensembles are generated. We have emphasized that they are "sampling-based" in the revised manuscript. |
| P9 L18 | Agree. An important point. | We are glad to hear that the reviewer agrees that this paradigm of modelling is still a niche. |
| P9 L23 | As suggested above a question about sensitivity analysis would have helped. Here a quote from Majone, G. (1989). Evidence, argument, and persuasion in the policy process. Yale University Press. Retrieved from https://yalebooks.yale.edu/book/9780300052596/evidence-argument-and-persuasion-policy-process "Are the results from a particular model more sensitive to changes in the model and the methods used to estimate its parameters, or to changes in the data?" | Thanks for the comment and the reference. If we can run such a survey again, we will include a question on sensitivity analysis, too. |
| P10 L14 | Sampling to generate scenarios? I would add that scenarios are also and perhaps primarily the result of a process of negotiation. The authors use systematically the word scenario and the expression never policy options. | We agree that the word scenario also refers to "policy scenarios" in the literature. In this manuscript, however, we refer to the model-based scenarios that are automatically generated by sampling. Each scenario refers to a computational experiment as defined in Page 3, Line 37. To clarify, we have added "sampling-based" there. We agree that not only policy scenarios, but also what-if scenarios that explore uncertainties can be the result of a negotiation process. However, we would like to refrain from mentioning such hybrid scenarios, i.e. the scenarios generated by both a model and by stakeholders qualitatively, since our objective is to focus on quantitative models. |
| P13 L6 | Perhaps here one should just accept the null hypothesis. | We have revised the text, both in the main manuscript and in the Methods section, to clarify that there is no strong statistical evidence for such a dependence when p<0.1. Still, we mention the finding with p<0.1 to discuss a potential relationship between the responses and experience level, not to claim dependence. |

## Reviewer 3

We are thankful for your careful and very constructive review of our manuscript. As you stated, the objective of this manuscript was to provide an indication of the status-quo, and we hope that it will receive interest.

We understand your scepticism about the word clouds. We have provided the data, i.e. the word frequencies, underlying these figures in the supplementary material in a tabular form.

The data and the analysis scripts (for both text-mining and the survey) are publicly available in an online repository as stated in the Code and Data Availability section. We had not report the repository address in the original manuscript since it reveals the author identity.

We understand why survey design could be problematic. In the Methods section of the revised manuscript, we have more extensively discussed the survey design, and the reasons for the choices we made.

To clarify the contribution of the paper, we have restructured the Discussion section. We highlight our key findings, not only related to data-orientation, but to many dimensions of the analysis, such as the decision-makers’ view and the validation in the context of scenario generation. We have also extended the practice and future research suggestions based on the findings.

Our responses to your specific comments can be found in the table below.

| **Place in the Orig. Manuscript** | **Comment** | **Response** |
| --- | --- | --- |
| P1 L21 | You talk about exploratory modelling later, but I think this dichotomy provides a false start to the topic. Modelling can be descriptive in different ways. Consolidative vs exploratory modelling are both descriptive, but fundamentally different. Similarly, hard systems science vs soft systems science models have long distinguished between modelling that attempts to capture (objective) reality vs a (subjective) point of view. Also, not all normative modelling frames its results as optimal decisions, only as "what should be done" A possible solution: "Some of these models have a normative stance, generating recommendations for action. Others aid decision making in other ways, while still capturing the complexity ..." | Thanks for a concrete suggestion to revise this part of the manuscript. There are surely various dimensions according to which models can be categorized. Here, the purpose was to distinguish between two broad categories of optimal control and dynamic simulation models, which provide temporal predictions. This was not intended to refer to exploratory models we discuss later. We have revised the text as suggested. |
| P1 L36 | Perhaps better to say "Validation is a modelling step commonly employed to ..." At the moment, it feels like validation has been singled out at the expense of uncertainty analysis, sensitivity analysis, robustness assessment (Edit: I note in the supplement that SA has been included in validation. This probably needs some clarification) More broadly, I think at this point it would be useful to give a very high level definition of validation that distinguishes it from other techniques for dealing with model credibility in decision making. p2 gets into definitions in more detail, but something is needed here already. In my own work, I've identified 18 different ways of framing the role of uncertainty that are in common use to deal with criticism. Concepts related to validation only form a small (but commonly used) part of this. | We have revised the text to emphasize that validation is "commonly" employed and "conventional" approaches focus on representation accuracy. However, we do not think that this statement implies, and we do not mean, that validation excludes uncertainty analysis. Instead, it means that validation includes dealing with uncertainties since there is also quite some criticism about the way uncertainties are dealt with. We have revised the text and clarify this at the end of the paragraph (P2 L2-5), where in alternative modelling paradigms, validity is established not by reducing uncertainty and but dealing with uncertainties differently.  Thanks for the reference on uncertainty framing. We think that it is a very useful evidence and resource to articulate the uncertainty communication problems. We use it explicitly to discuss our text-mining finding on the absence of uncertainty. |
| P2 L4 | "representation accuracy plays a less important role." It would be useful to explain why, e.g. "because the representation of the system is expected to be uncertain, and that uncertainty is dealt with by other means in decision making" | Thanks for the suggestion. We understand the importance of clarifying the reason for this statement. Therefore, we have revised the text as suggested. (P2 L2-5) |
| P2 L5 | "validation viewpoints on and approaches to general model validation practice" Delete first "validation"? | We have revised the text as suggested. (P2 L6) |
| P2 L6 | "when models are used specifically for scenario generation to acknowledge uncertainty" A little awkward "when models are used specifically to generate scenarios used to explore uncertainty"? | We have revised the text as suggested. Namely, we say "exploring multiple plausible futures", instead of "acknowledging uncertainty". (P2 L7-8) |
| P4 L12 | "The first observation on this body of literature is that the number of model validation publications has significantly increased over time." Is this simply because total number of publications has increased? Is the number of model validation publications increasing faster than total number of publications? Edit: Supplement 1 addresses this question, but shows large variation in % of validation-related publications. I would prefer to see a test that the increase is indeed statistically significant (it does look like it might be). I'm not sure I would call the increase substantial though, e.g. 1981, 1987, 1991 vs recent times. | This statement refers only to the total number of validation publications, and the following sentence complements it by clarifying that it is still a low fraction of the total modelling publications. In other words, yes, it is highly likely that the increasing trend is due to the increase in the number of academic publications in general. We have revised the main manuscript and the supplementary material to clarify that validation publications have been a small fraction "regardless of time". We, however, refrain from presenting a statistical test about this, since we do not aim to highlight this temporal trend as a main argument in the paper. |
| P5 L15 | "In both topics, uncertainty receives no attention," What do you mean by this? How would topic modelling have given it attention? Would you expect the word stem "uncertain-" to come up?  I assume the word is present in the corpus (even if it is only abstracts). Would changing any decisions in the analysis cause the word to appear? You might also want to comment/acknowledge factors that might lead to the word "uncertainty" not being used in the abstract (I touch on this issue in broad terms in the paper cited above) | This sentence is indeed based on the absence of the word stem "uncertain" in the topic words. To clarify, we have revised the text to say the figures depict the top 50 words (P5 L6), and "uncertainty is not among the top fifty words" in these topics (P5 L16). The word "uncertain" could indeed be present in the corpus, yet not coming up as a frequent word defining the Methods topic indicates that it is not explicitly and frequently used. We have also added a sentence that discusses this finding in light of the uncertainty framing findings in the paper you recommended (P5 L17-19). |
| P7 L13 | "would be expected to favour usefulness more than representation accuracy." However, this would be consistent with the notion that model users value models primarily for the insights gained as a result of their predictive or representation accuracy. i.e. if doesn't represent (something) well enough, then it's not going to be useful. Other questions might clarify whether this is the case? | In the manuscript, we discuss that usefulness is beyond representation accuracy, and we communicate this to the survey respondents prior to the questions. Therefore, we expect the respondents to evaluate this statement accordingly. Still, to be precise and clear, we have revised the manuscript (P7 L21-23) to say "model users... would be expected to ... favour usefulness more than representation accuracy, unless their purpose is an accurate representation." Furthermore, we have revised the manuscript earlier (P3 L13-23) to give a more clear description of usefulness. |
| P7 L17 | "Therefore, a dichotomy does not exist among practitioners" I don't think this can be concluded from the results shown. You would have to show results of the two questions combined. Perhaps the 20% who disagreed with Q1, fully agreed with Q2 Similarly 8% disagreeing with Q2 might fully agree with Q1. I would hypothesise three groups: focussed on reality, usefulness, and both The wording of the question is also ambiguous. Some respondents may have felt they were only allowed to choose one, while others may have felt permitted to say both are "the most important", given that the question is repeated. | This conclusion is based on the simultaneous responses given to the two questions, which we indeed did not discuss in the original manuscript. We now added a new figure to the Supplementary Information (Figure S.6) that shows the responses given to these two questions with respect to each other. In the main text (P7 L14-15), we clarify that this conclusion is based on simultaneous responses, too, and refer to the supplementary figure. |
| P7 L24 | "objections to using data oriented validation approaches due to the impossibility of a purely positivist validation are not widely acknowledged by practitioners." I don't think this statement is supported. It is quite possible for practitioners to acknowledge the objections but dismiss them as irrelevant to their context. I believe the paradox can easily be resolved from a sense-making perspective. Practitioners acknowledge that purely positivist validation is impossible, but within their work, it is still useful to act as if positivist validation were possible - it helps to make sense of what results to trust for a given purpose, and to convince others that one's research is worth paying attention to. Investment firms have to include the disclaimer that past results are not predictive of future performance precisely because they *can* be, and they are still *used* that way. Personally, I'm a pragmatic relativist - I find it very useful to appeal to positivist arguments in some circumstances.  I would be happy if this sentence was rephrased to say something like "objections to using data oriented validation approaches due to the impossibility of a purely positivist validation do not result in practitioners completely rejecting those approaches" | Thanks for reflecting on this finding also from a personal perspective, and for the paraphrasing. The intention of this sentence was to draw attention to the difference between the theoretical literature and the practice we try to cover in our analysis. The reasons for this difference could surely be beyond (not) acknowledging the impossibility of a purely positivist validation. We also agree that positivist validation is very important and helpful in many contexts. We do not claim otherwise. To clarify this, we have revised the text as suggested, namely avoiding the use of "acknowledgement". (P8 L11-13) |
| P8 L36 | "it can be said that the respondents favour a structure focused validation when the model purpose is scenario generation" It seems Q5 is the converse of Q3 (and it's nice to see this kind of check). However, the 79% that think model output importance <= model structure importance only translates to 40% who think that model structure importance => model output importance. Presumably the remaining 39% think they are equally important or are unsure, or were influenced by question wording. The point is not quite as unequivocal as this sentence makes it seem - a few extra words of nuance are needed. | We have revised the main text to say that the respondents do not favour an output-focused validation over a structure-focused one (P9 L23-29). This statement is more precise given the findings. In the supplementary information, we discuss the responses to these two questions in more detail, based on a new figure that shows the respective answers (Figure S.9). The majority of respondents who disagree with the output agree with the structure. Still, there are many respondents who disagree with the output yet remained neutral or disagree also with the higher importance of the structure. Therefore, these respondents can be said to value the output and structure almost equally, or to be indecisive about the answer. |
| P9 | "Many scholars have argued that data oriented validation is not sufficient, and that validation should include conceptual aspects and participatory approaches to enhance the public reliability of models. Our results show that this view has not been widely implemented," I don't think this point is sufficiently supported. You would need a specific analysis of whether conceptual aspects and participatory approaches are being used *in addition to* data-based methods. I would be happy with a wording in the direction of "Our results do not indicate wide implementation of this view" (i.e. you have absence of evidence, not yet evidence of absence) Edit: Figure S.5 clearly shows that using both data comparison and sensitivity analysis/reality checks is common. That seems to contradict the conclusion here. | We have revised the text as suggested (P10 L13-16), also to clarify that this conclusion is based on the text-mining results, which did not yield any findings related to conceptual and participatory approaches, but the prevalence of a data-driven validation. Our survey results indeed show that reality checks and sensitivity analyses are commonly used. These tests include 'conceptual aspects', yet still not 'participatory approaches'. |
| P9 L14 | "relative difficulty of defining usefulness compared to representativeness," I would say "relative difficulty of measuring ..." Defining representativeness is very difficult too, if you look deep enough into it. However, it is relatively easy to come up with many widely transferable indicators (e.g. see Bennett et al.) In contrast, usefulness is always obviously context-dependent, so any attempt to adopt a transferable indicator is likely to be controversial. The effort in measurement comes from having to understand the application context in sufficient depth (see e.g. Haasnoot et al.) for the measurement to be credible. The following sentence about resources needed already seems to imply this. | We agree that defining representativeness has difficulties, too, with so many technical aspects and different methods that can be considered. Still, we think that defining usefulness, in addition to measuring it, is difficult because this definition should be customized according to the purpose and the stakeholders involved. Therefore, we revise the text to include both "defining" and "measuring". (P11 L1) |
| P9 L18 | "since the academic literature vastly emphasizes prediction, and most practitioners think that models can be used for prediction purposes" I don't think this argument holds. As mentioned here and in Maier et al., an approach with multiple plausible futures is usually used in combination with a best-estimate approach. Different uncertainties in the model are dealt with in different ways. Therefore emphasis on prediction (in some form) will always exist. I do still agree that "multiple plausible futures" is a niche, but not based on any evidence presented here. | We agree that 'paradigms' discussed in Maier et al. are often used in combination. However, we do not think that 'best-estimate' and 'multiple-plausible futures' paradigms fundamentally align. They can surely be used in complementary manner, yet they are on different ends of a spectrum. We also agree that, regardless of the modelling paradigm, emphasis on prediction in some form can always exist. Therefore, we agree that having 'predict' in the text-mining results is not a sufficient evidence. However, since the prediction view is highly supported in the survey results, we combine the two to reach a conclusion about the multiple-plausible futures paradigm being a niche. |
| P9 L21 | "Yet, model structure is considered more important than the output in this context, as indicated by both the literature and practitioners" See comment above. I don't think this statement is 100% certain. | As discussed in response to an earlier comment, we have revised the text and added supplementary material about the survey responses on the relative importance of model structure and output in the context of scenario exploration. According to the results, the majority of respondents who disagree with a higher importance of output agree with a higher importance of structure. There are also many respondents who remain neutral or disagree with a higher importance of structure. We revised the text accordingly (P11 L33-36), yet based on this finding; we still propose a structure-oriented validation for future studies. As mentioned before, we provide a detailed discussion in the Supplementary Information (Figure S.9) |
| P13 L13 | "We reject the null hypothesis, ...., therefore we use p<0.1 to reject the null hypothesis of independence for these questions." This sounds a little suspicious. It might be good to give a different justification, or to explain the impact of this decision on results. | We have revised the text, both in the main manuscript (P9 L11-13) and in the Methods section, to clarify that there is no strong statistical evidence for such a dependence when p<0.1. Still, we mention the finding with p<0.1 to discuss a potential relationship between the responses and experience level, not to claim dependence. |
| Fig S.8 | "A higher percentage of the experienced respondents disagree that model validation should be performed differently when the model purpose is scenario generation." It seems to me that this is more a case of more experience=more confidence/stronger opinion. The results are still very divided. | We have revised the text in the supplementary material to clarify that disagreement with the corresponding statement is more common among the medium-level (2-10 years) experienced respondents. This figure is not labeled as Figure S.12. |

# **Reviewers’ Comments – Round 2**

**REVIEWER #2**

**General Comments**

a. Surprising that sensitivity analysis in presented only once, as a problem instead of as a

possible ingredient of a solution (P.4). A reference should be given for SA. I would suggest

the most quoted textbook, but the authors are free make a different choice: Saltelli, A.,

Ratto, M., Andres, T., Campolongo, F., Cariboni, J., Gatelli, D. Saisana, M., Tarantola, S., 2008,

Global Sensitivity Analysis. The Primer, John Wiley & Sons publishers. The Authors focus on

SA for model reduction, which is one of the many uses of SA, to note that model

reduction/parsimony has a trade off with a model’s capacity to surprise the analysis. This is a

well-known hurdle but hardly the first thing one might wish to say about SA. The authors are

invited to read without need to quote a recent reflection on the potential role of sensitivity

analysis in improving model quality assurance procedures here.

b. The Authors affirm (in the reply to reviewers) to have a sound knowledge of literature on

post normal science and derived methodologies. Perhaps they could mention the use of

models as metaphors, or models as an avenue for participatory exercises aimed at a

discovery of implicit norms and metaphors. See Ravetz, J. (2006). Models as metaphors.

Kasemir (ed.), Public Participation in Sustainability Science. Cambridge University Press,

available at

http://www.andreasaltelli.eu/file/repository/Models_Metaphors_with_Cover.pdf.

Incidentally pages 3-5 of the primer mentioned in (a) above put SA in the context of a post

normal science approach. Note that the Authors never mention post normal science in the

paper, while advocating “to evaluate the conceptualization, structure and behaviour of a

model with respect to its predefined purpose, based not only on in house testing but also on

peer reviews by experts and stakeholders” (P.11). In this context, perhaps, a mention of PNS

extended peer community concept could be useful.

**Specific Comments**

P.1 Start the Abstract with this sentence from Conclusions: This study investigated the viewpoints on

validation in the general modelling context and in the particular scenario exploration context.

P.1, Line 11 suggested revised text: … usefulness in relation to a given task is more important for

model developers

P.1, Line 12/13 suggested revised text: … they tend to better acknowledge the decision makers’

demand for clear communication of assumptions and uncertainties.

P.1, Line 21/22 suggested revised text: … Some of these models have a prescriptive stance,

generating recommendations for action. [Note: All models have normative stances, be these implicit

or explicit. Prescriptive is more precise in this context]

P.2, Line 1, add perhaps reference [7] to references [14-18]

P.2, Line 4/5 suggested revised text: … and validation does not aim to reduce this uncertainty, but to

dealt with it by other means.

P.2, Line 7, delete “, and”

P.2, Line 33/34 suggested revised text: … The relativist validation approaches value the model

usefulness – in the sense of fitness for purpose - more than the representation accuracy …

P.3, Line 12/13 suggested revised text: …The impossibility of an accurate data-driven validation leads

to defining model usefulness in terms of its fitness for a given purpose

P.4, Line 9/10, suggested revised text: Sensitivity analysis (Saltelli et al., 2008) can go a long way to

assist in model validation, and can be a useful ingredient even in participatory settings to make sure

e.g. that uncertainty has not be instrumentally inflated or deflated [7]. Often sensitivity analysis is

used for model simplification – in this case it can reduce model’s ability to surprise the analyst.

P.7, Lines 21/23, suggested revised text: This finding is counterintuitive, considering that model

users, either in research or decision-making contexts, would be expected to value how well the

model serves for its purpose and favour usefulness more than representation accuracy, unless their

purpose is an accurate representation. In fact, users expect from models more reality than model

can provide, an asymmetry well noted in [32]. [Authors: this is the point as made by [32] Jakeman

and al. “It is easy for a poorly informed non-modeller to remain unaware of limitations,

uncertainties, omissions and subjective choices in models. The risk is then that too much is read into

the outputs and/or predictions of the model.”]

P. 10/11 paragraph across the two pages; suggested rephrasing: Usefulness as fitness for purpose

might offer a guidance for model validation, but this view does enjoy as much support as one might

aspect. This might be due to the prevailing perception of models as description of the real,

entrenched among modelers as well as among model users. Additionally, the lack of resources to

engage experts, stakeholders and decision makers in the validation practice can make the fitness

approach more difficult to pursue.

P. 11, Line 3, references 33 and 35.

P. 11 Lines 20/25 The Author have rediscovered (no offense) the need for the extended peer

communities in model validation.

P. 12, Line 9/10, suggested revised text: Therefore, model validation should include an appreciation

of these attributes in the case where the scenario ensemble is generated by an exploratory model.

P. 12, Line 11/12, suggested revised text: Plausibility, consistency and diversity could then become

part of the assessment criteria, and techniques could be developed to [Authors to complete as the

original sentence was unclear]

**REVIEWER #3**

Thank you for thorough responses and revisions
The authors have effectively clarified some key points, and moderated problematic claims without detracting from them.
I recommend publication as-is.

 **REVIEWER #4**

This paper provides a very useful insight into the approaches taken when validating models for use in decision making and resource management. Particularly the authors provide a very good description of the different perspectives model users and developers take when considering how to validate a model and when a model will be classed a useful. Overall, I think the paper is well written and should be accepted for publication after the following has been addressed.

My only remaining concern is the discussion in relation to the final point raised by the reviewer regarding the evaluation of scenario ensembles. The original reviewer raised the issue that there are existing frameworks to evaluate model ensembles. In response the authors indicate that they are referring to models used to generate scenario ensembles with the multiple plausible futures paradigm. It is here I feel that the authors could expand on there discussions a little further and discuss model evaluation frameworks such as the GLUE analysis and the equifinality concept (Beven 2006). The nature of the GLUE methodology aims to account that multiple model realisations can provide successful validation against whatever measure of validation is chosen by the model user. More importantly GLUE can be used to propagate associated uncertainties through the generation of multiple possible future scenarios (as defined by the authors themselves in the text). Admittedly the uncertainties are often conditioned on the performance against past data however they can be used to provide some quantification of model evaluation/validation. I believe that techniques such as GLUE may be what the reviewer was referring to here and should at least be referred to in the discussion.

# **Response to Reviewers – Round 2**

## Reviewer 2

We are thankful for your second review on our manuscript. We have revised the manuscript following your suggestions. Below are our responses to your general comments as well as the minor comments.

Regarding your first general comment about sensitivity analysis, we admit that a reference to sensitivity analysis was missing, because we expected anyone in the modeling field to be familiar with this step of modelling. We have now included a reference to (Saltelli et al., 2008) for sensitivity analysis. Regarding the role of sensitivity analysis in validation, we have revised the text in Page 4 Lines 16-22, incorporating your specific suggestion. Specifically, we clarified why the use of sensitivity analysis in validation (references 21 and 53), as a part of the modelling cycle, can lead to undesired robustness in the case of exploratory modelling.

Regarding your second general comment about post-normal science, thanks for the suggestion to use Ravetz (2003) for the idea of using models as metaphors. We think that this aligns with the discussion we presented in Page 3, Lines 21-32 about using models as heuristics. We have inserted the use of models as metaphors in participatory settings as an alternative in Lines 27-29 in that paragraph. About involving peer communities in validation, we earlier mentioned the use of participatory approaches in validation (P3, Lines 3-4), including references from the PNS literature.

| **Place in the Original Manuscript** | **Comment** | **Response** |
| --- | --- | --- |
| P1 | Start the Abstract with this sentence from Conclusions: This study investigated the viewpoints on validation in the general modelling context and in the particular scenario exploration context. | The manuscript guidelines of the journal (<https://www.nature.com/documents/ncomms-manuscript-checklist.pdf> ) ask for providing background information in the first 2-3 sentences of the Abstract. Therefore, we were not able to accommodate this suggestion. |
| P.1, Line 11 | suggested revised text: … usefulness in relation to a given task is more important for model developers | We have inserted "regarding a given task" into the corresponding sentence. (P1 L11) |
| P.1, Line 12/13 | suggested revised text: … they tend to better acknowledge the decision makers’ demand for clear communication of assumptions and uncertainties. | The text has been revised as suggested. (P1 L12) |
| P.1, Line 21/22 | suggested revised text: … Some of these models have a prescriptive stance, generating recommendations for action. [Note: All models have normative stances, be these implicit or explicit. Prescriptive is more precise in this context] | The text has been revised as suggested. (P1 L22) |
| P.2, Line 1 | add perhaps reference [7] to references [14-18] | The reference has been added. (P2 L3) |
| P.2, Line 4/5 | suggested revised text: … and validation does not aim to reduce this uncertainty, but to dealt with it by other means. | The text has been revised as suggested. (P2 L6-7) |
| P.2, Line 7 | delete “, and” | We would like to keep ", and" since it refers to the second context we are addressing in this study, i.e. the scenario generation context. |
| P.2, Line 33/34 | suggested revised text: … The relativist validation approaches value the model usefulness – in the sense of fitness for purpose - more than the representation accuracy | The text has been revised as suggested. (P2 L36-37) |
| P.3, Line 12/13 | suggested revised text: …The impossibility of an accurate data-driven validation leads to defining model usefulness in terms of its fitness for a given purpose | The suggested revision could lead to a slightly different meaning, because we do not refer to a different definition of usefulness, but to a usefulness view as opposed to a positivist view. Therefore, we have revised the text as "...leads to the usefulness view in validation, i.e. where usefulness can be defined as how well a model fits for a given purpose" (P3 L19-20) |
| P.4, Line 9/10 | suggested revised text: Sensitivity analysis (Saltelli et al., 2008) can go a long way to assist in model validation, and can be a useful ingredient even in participatory settings to make sure e.g. that uncertainty has not be instrumentally inflated or deflated [7]. Often sensitivity analysis is used for model simplification – in this case it can reduce model’s ability to surprise the analyst. | We have revised the text to clarify the role of sensitivity analysis in validation, incorporating your suggestion in the general comments. (P4 L16-22) |
| P.7, Lines 21/23 | suggested revised text: This finding is counterintuitive, considering that model users, either in research or decision-making contexts, would be expected to value how well the model serves for its purpose and favour usefulness more than representation accuracy, unless their purpose is an accurate representation. In fact, users expect from models more reality than model can provide, an asymmetry well noted in [32]. [Authors: this is the point as made by [32] Jakeman and al. “It is easy for a poorly informed non-modeller to remain unaware of limitations, uncertainties, omissions and subjective choices in models. The risk is then that too much is read into the outputs and/or predictions of the model.”] | Thanks for drawing our attention to this statement in Jakeman et al. (2006). We are glad to hear that our findings support this argument. We have revised the text to include this reference and argument. (P8 L17-20) |
| P. 10/11 | paragraph across the two pages; suggested rephrasing: Usefulness as fitness for purpose might offer a guidance for model validation, but this view does enjoy as much support as one might aspect. This might be due to the prevailing perception of models as description of the real, entrenched among modelers as well as among model users. Additionally, the lack of resources to engage experts, stakeholders and decision makers in the validation practice can make the fitness approach more difficult to pursue. | We have revised the text in the corresponding paragraph to incorporate the suggestion. (P11 L18-23) |
| P. 11, Line 3 | references 33 and 35. | Earlier in the manuscript (Page 2, Line 2), several references are cited for integrated validation approaches, including 33. For this sentence, we prefer to refer to 33 only, which focuses on "model validation" and provides a clearer overview of the subject. |
| P. 11 Lines 20/25 | The Author have rediscovered (no offense) the need for the extended peer communities in model validation. | This is an interpretation of the findings, which surely relate to consulting extended peer communities in validation, as we discuss earlier (Page 3, L 3-4). We are happy to reconsider this discussion if the reviewer clearly recommends a change. |
| P. 12, Line 9/10 | suggested revised text: Therefore, model validation should include an appreciation of these attributes in the case where the scenario ensemble is generated by an exploratory model. | Thanks for the suggestion. We have revised the text exactly as suggested. (P12 L23-24) |
| P. 12, Line 11/12 | suggested revised text: Plausibility, consistency and diversity could then become part of the assessment criteria, and techniques could be developed to [Authors to complete as the original sentence was unclear] | We have revised the text accordingly, also taking into account the comment of Reviewer 4 about the evaluation of scenario ensembles. (P12 L22-29) |

## Reviewer 3

Thanks for your assessment and final suggestion for publication. We think that the paper has significantly benefited from your review.

## Reviewer 4

Thanks for your assessment and positive remarks. We are glad to receive this suggestion about the GLUE analysis. We have incorporated it into the Model Validation section as an alternative approach to address the equifinality principle (Page 3 Lines 13-18). In the discussion section (Page 12, Line 22-29), we have clarified the discussion on the evaluation of scenario ensembles, including new references to the scenario attributes.

# **Reviewers’ Comments – Round 3**

**REVIEWER #2**

No more comments from my side.


**REVIEWER #4**

I would like to thank the authors for their detailed responses to my original concerns regarding the scenario ensembles and the expansion of the discussion on model evaluation techniques. I am happy that these concerns have now been addressed and therefore recommend publication as is. This paper provides a very interesting insight into approaches taken when validating models and will be a very useful contribution to the literature - well done!
